# Assessment of seafood contamination under the marine strategy framework directive: contributions of the German environmental specimen bank

**DOI:** 10.1007/s11356-018-2728-1

**Published:** 2018-07-14

**Authors:** Annette Fliedner, Heinz Rüdel, Burkhard Knopf, Nina Lohmann, Martin Paulus, Michael Jud, Ulrike Pirntke, Jan Koschorreck

**Affiliations:** 1 Fraunhofer Institute for Molecular Biology and Applied Ecology (Fraunhofer IME), Department Environmental Specimen Bank and Elemental Analysis, 57392 Schmallenberg, Germany; 2 Eurofins GfA Lab Service GmbH, Neulaender Kamp 1a, 21079 Hamburg, Germany; 3 Department of Biogeography / Environmental Specimen Bank, Trier University, 54286 Trier, Germany; 4 Federal Office of Consumer Protection and Food Safety (BVL), Unit 101, P.O. Box 110260, 10832 Berlin, Germany; 5 German Environment Agency (Umweltbundesamt), 06813 Dessau-Rosslau, Germany

**Keywords:** Marine strategy framework directive, Descriptor 9, Biota monitoring, Environmental specimen bank, Blue mussel, Eelpout

## Abstract

Descriptor 9 (D9) of the European Union Marine Strategy Framework Directive refers to the compliance of contaminant levels in fish and other seafood of a defined marine region or subregion with human health threshold values. This requires georeferenced samples that are often difficult to obtain when relying on commercial fisheries or programs designed for monitoring human exposure. The present study examines whether georeferenced samples of blue mussel (*Mytilus edulis*) and eelpout (*Zoarces viviparus*) fillet of the German environmental specimen bank (ESB) can be used in this context. The suitability of the ESB samples, procedures, and analytical methods is evaluated with respect to D9 requirements. Based on ESB data for the D9 relevant contaminants Pb, Cd, Hg, ∑4 PAHs, PCDD/Fs, dioxin-like (dl)-PCBs, and indicator non-dl-PCBs and the potentially relevant substances TBT, PFOS, PBDE, and HBCDD, the Good Environmental Status for D9 is assessed at the ESB sites in the North and Baltic Seas. The overall evaluation indicates that ESB samples are suitable for D9 assessment with the limitation that only coastal areas of the North and Baltic Seas are covered. Over a period of up to 30 years, concentrations of the D9 relevant contaminants were well below the maximum levels allowed for human consumption.

**Electronic supplementary material:**

The online version of this article (10.1007/s11356-018-2728-1) contains supplementary material, which is available to authorized users.

## Introduction

Marine environments are under massive pressure caused by anthropogenic exploitation and pollution. This includes pollution with chemical substances and marine litter, extensive fishing activities, deterioration of the sea floor, e.g., by construction measures, extraction of minerals, and fishing with ground nets, and introduction of noise, e.g., by ships, construction, renewable energy, and tourism (Ban and Alder 2008; Boldt et al. 2014; Halpern et al. 2008; IOC/UNESCO et al. 2011). As a consequence, the biodiversity has decreased, fish stocks have declined, and the resilience of marine ecosystems has dwindled in many parts of the world (e.g., Caddy and Seijo 2005; Costello et al. 2010; Johnston and Roberts 2009; Lotze et al. 2011; Pauly et al. 2005). Acknowledging this, the European Union has adopted the Marine Strategy Framework Directive 2008/56/EC (MSFD) (EC 2008a, and its amendment Commission Decision (EU) 2017/848 (EC 2017a)) that aims at the conservation and protection of the EU marine waters.

By 2020, a Good Environmental Status (GES) of the marine environment shall be achieved whereby the GES is defined as “the environmental status of marine waters where these provide ecologically diverse and dynamic oceans and seas which are clean, healthy and productive within their intrinsic conditions, and the use of the marine environment is at a level that is sustainable, thus safeguarding the potential for uses and activities by current and future generations…” (EC 2008a). This definition implies an ecosystem-based approach of assessment that considers structure and function of marine ecosystems (Borja et al. 2013; Walmsley et al. 2017).

To assess the status of the ecosystems, the MSFD requires 11 descriptors (D) that address different elements of the marine environment. D1 and D4 relate to the state of the ecosystem, i.e., its biodiversity and food webs, while the other descriptors are pressure-related addressing issues like non-indigenous species (D2), commercial fishing (D3), eutrophication (D5), physical loss and disturbance of the seafloor (D6), hydrographical changes (D7), contamination (D8, D9), marine litter (D10), and energy and underwater noise (D11). Each of these qualitative descriptors is broken down to quantifiable criteria. In total, 42 criteria have been specified and standardized methods for monitoring and assessment have been defined (Borja et al. 2011; EC 2017a).

Descriptors D8 and D9 both deal with contaminants. D8 refers to contaminants in marine water, sediment, or biota which are assessed against threshold values (i.e., values set in accordance with Water Framework Directive WFD (EC 2000) and its daughter directives (EC 2008b, 2013a) or, if not applicable or no value is set under the WFD, values set by Member States through regional or subregional cooperation) (EC 2017a).

Descriptor 9 focuses on contaminants in fish and other seafood for human consumption. The number of contaminants assessed under D9 is lower compared to D8 and comprises mainly those for which regulatory levels for foodstuffs are set under Regulation (EC) No 1881/2006 and its amendments (EC 2006a, 2008c, 2011a, b, c). However, on the basis of risk assessments, Member States can choose to not consider contaminants and/or include additional contaminants, for which threshold values must then be established by the Member States through regional or subregional cooperation (EC 2017a).

There is a link between Descriptors 8 and 9 (Gago et al. 2014; Law et al. 2010; Maggi et al. 2014; Swartenbroux et al. 2010; Walmsley et al. 2017; Zampoukas et al. 2012, 2014): Because many contaminants are transferred along the food web those of concern to marine fish will likely also be of concern to humans (Fleming et al. 2006). On the other hand, concentrations exceeding the regulatory levels for food will probably also affect the ecosystem because food regulatory levels are usually higher than thresholds for assessing environmental pollution (Swartenbroux et al. 2010).

Monitoring seafood related to human health is different from monitoring biota for environmental purposes: For the latter, a high degree of standardization and geographical traceability of the samples are crucial to the derivation of temporal trends and the assessment of compliance with reference values. In contrast, monitoring of seafood contamination for human consumption relies on the edible fraction of a wide variety of commercially relevant species for which the precise origin is not relevant and often unknown (Gago et al. 2014; Swartenbroux et al. 2010). The MSFD, however, requires that the GES has to be achieved or maintained for a specified region or subregion. The species monitored in the context of D9 shall be “relevant to the marine region or subregion concerned” (EC 2017a) implying that the geographical origin of the samples should be known.

The MSFD relies on the institutional structures and programs of existing Regional Sea Conventions to minimize monitoring costs and efforts, namely on the Convention for the Protection of the Marine Environment in the North-East Atlantic (OSPAR 1992), the Convention on the Protection of the Marine Environment in the Baltic Sea Area (HELCOM 1992), the Convention for the Protection of Marine Environment and the Coastal Region of the Mediterranean (UNEP-MAP 1995), and the Convention for the Protection of the Black Sea (Bucharest Convention 1992).

However, neither OSPAR nor HELCOM have so far developed indicators for the assessment of the status of fish and shellfish contamination in relation to human health as required by D9 (HELCOM 2017a; OSPAR 2017). In Germany, no marine monitoring programs exist that routinely collect georeferenced samples of edible fish in coastal waters and the German Exclusive Economic Zone (EEZ). A systematic assessment of fish addressing the specific requirements of MSFD is thus not possible to date. The recent assessment of the status of the marine environment in German waters under the MSFD mainly relies on samples from mussels in national waters and consequently does not fully address the requirements of D9 (BMUB 2018a, b).

Against this background, the question arises of whether other monitoring programs can step in and provide data that can help assess D9. In Germany, the German Environmental Specimen Bank (ESB) collects and archives environmental specimens from defined terrestrial, limnetic, and marine ecosystems since the mid-1980s (www.umweltprobenbank.de/en). All samples are analyzed for a set of classical contaminants (Rüdel et al. 2010). Meanwhile, long time series are available highlighting, e.g., effects of regulatory measures. Furthermore, the archived samples allow to retrospectively analyze additional chemicals of concern that come into focus (e.g., Rüdel et al. 2003, 2011). In the North and the Baltic Seas the ESB routinely collects bladder wrack (*Fucus vesiculosus*), blue mussels (*Mytilus edulis*), eelpout (*Zoarces viviparous*), and eggs from sea gulls (*Larus argentatus*).

With respect to D9 especially blue mussels and eelpout may be of interest. All samples are georeferenced and the archive allows the integration of new contaminants into the assessment or when time trends are required. However, regardless of the high operating standards of the ESB, it remains to be proven whether the nature of the samples and their handling and analysis are compatible with the requirements of the D9 monitoring under the MSFD.

The aim of the present study is to evaluate the suitability of the ESB samples for D9 assessment and, based on ESB data, to exemplarily assess D9 in the coastal marine regions of Germany whereby not only those contaminants are considered that are currently assessed under D9 but also substances that may be relevant for D9 assessment in the future.

## Material and methods

### Sampling sites

**Fig. 1:**
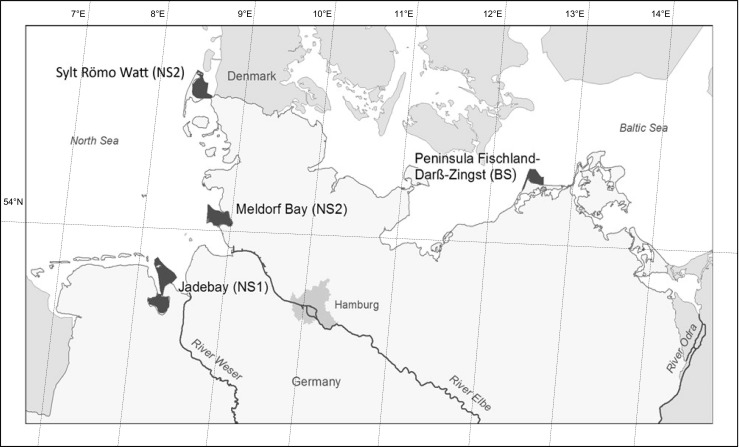
Marine sampling sites of the German Environmental Specimen Bank

The marine sampling sites of the ESB (Fig. 1) are located in the coastal areas of the Central North Sea (FAO/ICES Division 27.4.b) and the Baltic Sea West of Bornholm (FAO/ICES Subdivision 27.3d.24). The sites are clearly defined and georeferenced and partly extend beyond the one nautical mile zone. More details are given in Rüdel et al. (2003) and at www.umweltprobenbank.de/en.

The two North Sea (NS) sampling areas are part of the National Park Wadden Sea, more precisely of the National Parks and Biosphere Reserves “Lower Saxony Wadden Sea,” and “Schleswig-Holstein Wadden Sea” that are listed as UNESCO World Heritage sites. The areas are extensively exploited by fishery and subject to riverine inputs of hazardous substances via the rivers Elbe, Weser, and Ems.

In the Lower Saxony Wadden Sea (referred to as NS 1), the sampling site for mussels is located at the outlet of the Jade bight. Eelpout are fished in the Varel-Mellum Transect which extends over the entire tideway of the Jade Bight. Both sampling sites are influenced by emissions from agriculture and industries. Further contaminations stem from dumping and injection of dredged material from the Weser.

In the Schleswig-Holstein Wadden Sea (referred to as NS 2), mussels are collected in a bay at the north end of the island Sylt which is part of the Sylt-Römö Wadden Sea. At low tide, only a small tideway connects the bay with the Sylt-Römo Wadden Sea, while about three quarters of the bay are intertidal. Contamination in this area originates mainly from atmospheric deposition. In contrast to the rather protected mussel sampling site, the sampling area for eelpout is located further south in the Meldorf Bay that is part of the Germany Bight and opens to the North Sea. It lies directly north of the Elbe estuary and is exposed to a large spectrum of contaminants carried by the Elbe. Eelpouts are fished in the sublitoral areas of gat Piep that drains the Meldorf Bay.

The Baltic Sea sampling site (referred to as BS) for mussels and eelpout is located in the Bodden National Park of Western Pomerania which extends along the coast of Western Pomerania and includes several islands and the peninsula Fischland/Darß/Zingst. The national park is part of the world’s largest brackish water habitat and is extensively used by fishery and tourism.

Both mussels and eelpout are sampled at Darßer Ort, the northernmost point of the Fischland/Darß/Zingst peninsula. The sampling area covers the coastal area up to two nautical miles off Darßer Ort. Contaminations in this area stem mainly from agriculture, atmospheric deposition, and ship traffic.

### Sampling and processing

Sampling and processing under the German ESB program is highly standardized and follows the Standard Operating Procedures (SOPs) of the ESB (www.umweltprobenbank.de/en). Sampling and biometric characterization is performed by the ESB project team of Trier University. The entire process is accredited according to EN ISO/IEC 17025.

Blue mussels are collected every 2 months at both North Sea sites and twice per year in the Baltic Sea (June and November). Directly after sampling, the mussels are weighed and deep-frozen at < − 130 °C. In the lab, the soft bodies are carefully removed without allowing the samples to thaw. The respiratory water and intestinal content remain in the mussel as part of the sample, whereby the amount of respiration water is recorded (Paulus et al. 2018) (Table S1, Supplementary material).

Eelpout is sampled once per year in early summer (May–June) before the mating season to avoid any bias caused by reproductive activity, e.g., through transfer of lipid-associated contaminants to the gonads and eggs (Greenfield et al. 2005). Immediately after sampling, the fish are biometrically characterized and dissected under clean air conditions. The liver and both skinless fillets are separately deep-frozen at < − 130 °C (Klein et al. 2018) (Table S1, Supplementary material).

In the laboratory, the tissues are cryo-milled to a homogenous powder and pool samples are prepared. All eelpout fillets (or livers) and all blue mussel soft bodies sampled in 1 year at one site are pooled resulting in one annual pool sample per specimen and sampling site. Aliquots of the samples are stored in the ESB archive at temperatures below − 150 °C in an inert atmosphere (from evaporating liquid nitrogen coolant) to minimize chemical alterations (for details see Rüdel et al. 2003, 2010). Since the pollutants covered here are assessed as persistent, no concentration changes even after long storage periods are expected under these conditions.

### Chemical analysis

Chemical analysis of metals and tributyltin (TBT) compounds was performed at Fraunhofer IME, Schmallenberg. Organic compounds were analyzed by Eurofins GfA Lab Service, Hamburg. All methods are documented in laboratory-specific SOPs. Both laboratories have implemented quality assurance/quality control (QA/QC) measures. Table S2a–e (Supplementary material) summarizes the relevant QA/QC parameters.

Metal analyses follow the SOPs of the ESB (www.umweltprobenbank.de). Detailed descriptions are also given in Rüdel et al. (2010). Lead (Pb) and cadmium (Cd) were measured by ICP-MS, mercury (Hg) was determined using a Direct Mercury Analyzer (DMA-80, MLS, Leutkirch, Germany). Metal data were not corrected for recovery (no extraction but digestion of sample material; digestion efficiency confirmed by recovery data of appropriate certified reference materials).

Trace levels of TBT in current biota samples were analyzed after digestion, derivatization, and extraction by a validated sensitive SID-GC/ICP-MS method (Species Specific Isotope Dilution-Gas Chromatography-Inductively Coupled Plasma-Mass Spectrometry). Details are described in Radermacher (2015). Samples from years before 2009 were analyzed by gas chromatography applying an atomic emission detector (Rüdel et al. 2009). TBT data were not corrected for recovery.

Dioxins and furans (PCDD/F) and polychlorinated biphenyls (PCB) were analyzed by high-resolution gas chromatography followed by high-resolution mass spectrometry (HRGC/HRMS) using the isotope dilution method. Polybrominated diphenylethers (PBDE), benzo[a]pyrene (B[a]P), benzo[a]anthracene, (B[a]A), benzo[b,j,k]fluoaranthene, and chrysene (analyzed together with triphenylene) were determined by gas chromatography coupled to mass spectrometry (GC/MS). Quantification was carried out using isotope-labeled internal standards. Response factors were taken into account thus considering a correction of data for extraction efficiency. For more details, see Fliedner et al. (2016).

Hexabromocyclododecane (HBCDD, reported here as sum of α-, β-, and γ-diastereomers) and perfluorooctane sulfonic acid (PFOS) were analyzed by means of liquid chromatography coupled to mass spectrometry (LC/MS-MS). Quantification was carried out using isotope-labeled internal standards. Response factors were taken into account thus considering a correction of data for extraction efficiency. For more details, see Fliedner et al. (2016).

Quantification of the target compounds included the use of either internal or external standards. If available, isotope-labeled standards were applied. During extraction, clean-up and measuring method blanks were analyzed in parallel to each batch of samples (at least one method blank per 12 samples). Precision and accuracy were monitored along with each batch of samples by analyzing in-house QA matrix samples, sample material of previous interlaboratory proficiency studies, or certified reference material.

### Data treatment

In accordance with the MSFD (EC 2008a) and Reg. (EC) No.1881/2006 (EC 2006a), all data are expressed on a wet weight (ww) basis. Concentrations of PCDD/Fs and dl-PCBs are given as “WHO toxicity equivalents” (TEQ) that combine the toxicity of a defined set of dioxins, furans, and dioxin-like PCB (Van den Berg et al. 2006). Data are expressed as upper bound concentrations, i.e., the calculation includes the respective concentrations of the limits of quantification (LOQs) for congeners below or equal to the limit of quantification (LOQ).

Differences between contaminant concentrations at different sites were compared using the Mann-Whitney test (VassarStats 2018, http://vassarstats.net/index.html). Temporal trends were analyzed using the MS-EXCEL-based software tool “LOESS Trend” (Version 1.1) developed by J. Wellmitz (German Environment Agency). The tool fits a locally weighted scatterplot smoother (LOESS; fixed window width of 7 years) through the annual contaminant data followed by tests for significance of linear and non-linear trend components by means of an analysis of variance (ANOVA) (Fryer and Nicholson 1999). Based on the LOESS function and a *t* test, the tool also allows to determine the significance of differences between selected time points and the end of the time series provided that the time span in between is at least 7 years (contrast test).

## Results and discussion

### Consistencies and differences between MSFD requirements and ESB standards

The suitability of the ESB samples for D9 assessment was evaluated with respect to the MSFD requirements. Table 1 summarizes the main results. More details are given in Tables S1 and S2 (Supporting Information).

**Table 1:** Compliance of blue mussel (*Mytilus edulis*) and eelpout (*Zoarces viviparous*) samples from the German Environmental Specimen Bank (ESB) with requirements for assessing Descriptor 9 of the Marine Strategy Framework Directive (MSFD)

MSFD requirements regarding	Compliance with the MSFD requirements		Remarks
	ESB mussels	ESB eelpout	
Geographical scope	(x)	(x)	EEZ not covered
Temporal scope	x	(x)	Worst case for eelpout
Species	x	(x)	Eelpout are not among the most caught or consumed species
Sampling	(x)	(x)	Personnel not authorized according to Reg. (EC) No. 333/2007
Sample processing	(x)	x	Breathing water included in mussel samples
Analyzed contaminants	x	x	
Chemical analysis	(x)	(x)	ESB method analyzes benzo[b,j,k]fluoranthene and chrysene / triphenylene as co-elutions

*x* fulfilled, (*x*) partly fulfilled, *EEZ* exclusive economic zone

### Geographical and temporal scope

D9 monitoring requires that “the temporal and geographical scope of sampling is adequate to provide a representative sample of the specified contaminants in fish and other seafood in the marine region or subregion” (EC 2017a). The ESB samples cover three coastal regions that are considered representative of German coasts. However, the fishing grounds in the open seas of the EEZ are not covered. With respect to the temporal scope, the ESB sampling of blue mussels meet the D9 requirements. In the case of eelpout, the samples are not representative for the whole year but may be considered as worst-case scenario with respect to lipophilic contaminants in fillets because sampling takes place prior to mating season and thus before body lipids are transferred to the reproductive tissue (Greenfield et al. 2005).

### Species

The MSFD does not predefine the fish or mussel species and sizes to be analyzed for D9 assessment. The only specifications are that the species has to (1) be relevant to the marine region, (2) fall under the scope of Reg. (EC) No. 1881/2006, (3) be suitable for the contaminants, and (4) be among the most consumed in the Member States or the most caught or harvested for consumption. An indicative list with species is included in Swartenbroux et al. (2010). Zampoukas et al. (2014) also recommends to consider the ability of the species to biomagnify specific classes of contaminants and to ensure that different trophic levels and habitats are represented.

Blue mussels meet all the above mentioned criteria: They are frequent inhabitants of the German coastal regions and are commercially exploited for human consumption. The mussels live attached to rocks, poles, and other hard substrates in intertidal areas. As filter feeders they are exposed to both water soluble and particle bound contaminants and are therefore widely used as bioindicators and chemical pollution monitoring species in coastal waters (review: Beyer et al. 2017).

The suitability of eelpout for D9 assessment is less obvious. Eelpouts are abundant demersal fish in the German coastal regions. They have low migratory behavior and are often used as coastal bioindicators for biological and chemical effect assessments (Hedman et al. 2011; HELCOM 2017b; OSPAR 2013). However, while they are a welcome by-catch in commercial fisheries, they are not specifically fished upon and are not among the most caught or consumed species in Germany (listed in Centenera 2014).

Nevertheless, the contamination of eelpout can give an indication of the contamination of some of the most consumed fish because of similarity of exposure and habitat. Eelpout feed mainly on bottom-dwelling organisms like snails, insect larvae, crustaceans, and eggs and fry of fish. Their trophic level (TL) is around 3.5 which makes them comparable to, e.g., plaice (*Pleuronectes platessa*, TL 3.2) and flounder (*Platichthys flesus*, TL 3.3; all TL data according to Froese and Pauly 2017). Like plaice and flounder, eelpouts live in close contact to the sediment and are exposed not only to contaminants in the water phase and to bioaccumulated contaminants via trophic transfer but also to substances bound to the sediment.

The suitability of eelpout for D9 assessment is supported by data from Karl and Lahrssen-Wiederholt (2009) and Karl et al. (2010) who analyzed PCDD/Fs, dl-PCBs, and ndl-PCBs in cod (*Gadus morhua*) and herring (*Clupea harengus*) from georeferenced sites in the North and Baltic Seas: After lipid normalization levels in eelpout, cod and herring differ by no more than a factor of 2.

### Sampling and sample processing

According to Commission Decision (EU) 2017/848, sampling for the assessment of the maximum levels of contaminants (D9) shall be performed in accordance with the quality standards required in food legislation laid down in Reg. (EC) No. 882/2004 (regarding the performance of controls to ensure compliance with feed and food law, animal health and animal welfare; EC 2004), Reg. (EU) No. 644/2017 (regarding sampling and analysis for the control of dioxins, dl-PCBs and ndl-PCBs; EC 2017b), and Reg. (EC) No. 333/2007 (regarding sampling and analysis of metals and B[a]P; EC 2007 amended by EC 2011d).

The ESB meets most requirements regarding sampling and analysis for the control of dioxins, dl-PCBs, ndl-PCBs, and metals or has even stricter standards. Details are given in Tables S1 and S2 (Supporting material).

However, sampling under the ESB is not performed by officially authorized personnel according to Reg. (EC) No. 333/2007 and Reg. (EC) No. 644/2017, and no controls according to Reg. (EU) No. 882/2004 are performed during sampling and processing.

In the case of blue mussels, it should also be kept in mind that ESB mussel samples include the breathing water which accounts for in average 58% of the mussel wet weight at NS 2 and 67% at NS 1 and BS. Accordingly, respective factors of 3 (NS 1), 2.4 (NS 2), and 3 (BS) should be considered when comparing mussel data with maximum levels (MLs).

### Analyzed contaminants and chemical analyses

D9 refers to contaminants in fish and other seafood for which regulatory levels have been set to protect human consumers. These are heavy metals (Pb, Cd, Hg), PAHs (benzo[a]pyrene, benzo[a]anthracene, benzo[b]fluoranthene, and chrysene), dioxins, furans and dioxin-like PCBs (PCDD/Fs + dl-PCBs), and non-dioxin-like PCBs (ndl-PCBs).

Currently, all D9-relevant contaminants are covered by the ESB. The substances are analyzed either in blue mussel or eelpout samples (or in both). For PAHs, however, the analytical method used within the ESB program might lead to an overestimation regarding benzo[b]fluoranthene and chrysene to which the regulatory levels apply (EC 2011c) because the ESB’s analytical method is not able to distinguish between benzo[b]fluoranthene, benzo[j]fluoranthene, or benzo[k]fluoranthene and between chrysene and triphenylene but can only provide information on benzo[b,j,k]fluoranthene and chrysene/triphenylene as co-elutions. This should be taken into account when evaluating the data.

The available ESB data are summarized in Table S3 (Supplementary material). The table also includes information on additional contaminants that might be of interest for future assessments.

With respect to chemical analyses, the ESB meets all relevant requirements regarding sensitivity, accuracy, repeatability, and reproducibility (Table S2, Supplementary material). Requirements regarding recovery rates are not necessarily fulfilled for the analysis of organic contaminants. Nevertheless, the reliability of data sets concerning the pertained organic contaminants is checked and ensured within each batch of samples by analyzing in-house QA matrix samples, sample material of previous interlaboratory proficiency studies, or certified reference material.

For PAHs, the analytical method used within the ESB-program might lead to an overestimation regarding benzo[b]fluoranthene and chrysene because additional substances are determined (i.e., benzo[j]fluoranthene, benzo[k]fluoranthene, and triphenylene).

### D9 assessment based on ESB data

Annual pool samples of blue mussels and eelpout are routinely analyzed for the D9 relevant contaminants. Fairly long time series dating back to the 1990s are available for heavy metals, ∑4 PAHs and B[a]P in mussels and for Pb, Hg, and ndl-PCBs in eelpout (Table S3, Supplementary material). PCDD/Fs + dl-PCBs are currently only determined in eelpout, and time series are relatively short comprising every second year between 2003 and 2015 as well as 2016 and 2017.

With respect to contaminants potentially subject to D9 assessment, TBT, and the WFD priority substances PFOS, PBDE, and HBCDD are included in the present study. For TBT in eelpout and mussels, the available time series end in 2013 (Radermacher 2015). PFOS, PBDE, and HBCDD data have so far been analyzed in archived eelpout samples from every second year between 2003 and 2015 as well as in samples from 2016 and 2017 (Table S3, Supplementary material).

Tables 2, 3, and 4 summarize the contaminant levels in blue mussels and eelpout fillet.

**Table 2:**
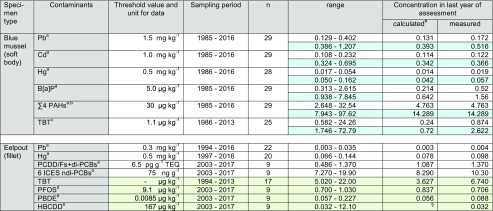
Contaminant concentrations in blue mussel and eelpout from the North Sea/ESB sampling site NS 1 (Lower Saxony Wadden Sea; FAO/ICES Division 27.4.b). White fields: D9 relevant contaminants; light blue shading: consideration of breathing water (measured concentration × 3); light green shading: additional contaminants not yet required for D9 assessment; concentrations for metals are given in mg kg^−1^, those for PCDD/Fs + dl-PCBs as pg g^−1^ WHO-TEQ, and all others in μg kg^−1^ (all wet weight based)

*n* number of annual pool samples, *n.s.* not significant

^#^Calculation based on linear trend analysis

^a^Maximum level for human consumption (Regulation (EC) No. 1881/2006 and amendments)

^b^Sum of benzo[a]pyrene, benzo[a]anthracene, benzo[b,j,k]fluoranthene and chrysene + triphenylene

^c^Wet weight Environmental Assessment Concentration (EAC) for mussels calculated from the dry weight EAC of 12 μg kg^−1^ and a mean water content of 90.6% in blue mussels from NS 1 during the sampling period

^d^Environmental Quality Standard (EQS) referring to protection goal “human health”

^e^EQS referring to protection goal “secondary poisoning”

^§^Value < 0, interpreted as not detected

**Table 3:**
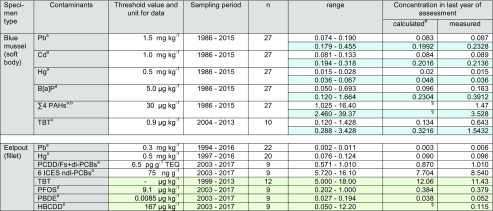
Contaminant concentrations in blue mussel and eelpout from the North Sea/ESB sampling site N2 (Schleswig-Holstein Wadden Sea; FAO/ICES Division 27.4.b). White fields: D9 relevant contaminants; light blue shading: consideration of breathing water (measured concentration × 2.4); light green shading: additional contaminants not yet required for D9 assessment, concentrations for metals are given in mg kg^−1^, those for PCDD/Fs + dl-PCBs as pg g^−1^ WHO-TEQ, and all others in μg kg^−1^ (all wet weight based)

*n* number of annual pool samples, *n.s.* not significant

^#^Calculation based on linear trend analysis

^a^Maximum level for human consumption (Regulation (EC) No. 1881/2006 and amendments)

^b^Sum of benzo[a]pyrene, benzo[a]anthracene, benzo[b,j,k]fluoranthene and chrysene + triphenylene

^c^Wet weight Environmental Assessment Concentration (EAC) for mussels calculated from the dry weight EAC of 12 μg kg^−1^ and a mean water content of 92.6% in blue mussels from NS 2 during the sampling period

^d^Environmental Quality Standard (EQS) referring to protection goal “human health”

^e^EQS referring to protection goal “secondary poisoning”

^§^Value < 0, interpreted as not detected

**Table 4:**
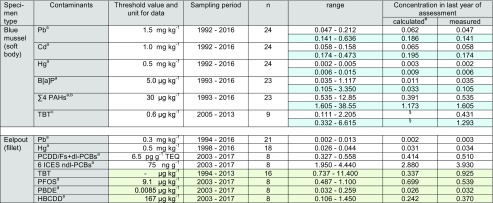
Contaminant concentrations in blue mussel and eelpout from the Baltic Sea/ESB sampling site BS (Bodden National Park of Western Pomerania; FAO/ICES Subdivision 27.3d.24). White fields: D9 relevant contaminants; light blue shading: breathing water considered (measured concentration × 3); light green shading: additional contaminants not yet required for D9 assessment; concentrations for metals are given in mg kg^−1^, those for PCDD/Fs + dl-PCBs as pg g^−1^ WHO-TEQ, and all others in μg kg^−1^ (all wet weight based)

*n* number of annual pool samples, *n.s.* not significant

^#^Calculation based on linear trend analysis

^a^Maximum level for human consumption (Regulation (EC) No. 1881/2006 and amendments)

^b^Sum of benzo[a]pyrene, benzo[a]anthracene, benzo[b,j,k]fluoranthene and chrysene + triphenylene

^c^Wet weight Environmental Assessment Concentration (EAC) for mussels calculated from the dry weight EAC of 12 μg kg^−1^ and a mean water content of 94.7% in blue mussels from BS during the sampling period

^d^Environmental Quality Standard (EQS) referring to protection goal “human health”

^e^EQS referring to protection goal “secondary poisoning”

^§^Value < 0, interpreted as not detected

Concentrations in the last year of sampling are given as calculated values (derived from the trend line) and as measured values. In some cases, these values differ significantly (e.g., for TBT by factors of 3.6 and 4.8 in mussels from NS 1 and NS 2, respectively, and for B[a]P by a factor of 3.2 in mussels from BS) indicating that the trend function may not adequately reflect the last year. In these cases, assessing compliance for the last sampling year should consider also the measured concentrations. Note that for ∑4 PAHs in blue mussels from NS 2, TBT in mussels from BS, and HBCDD in eelpout from NS 1 and NS 2, the calculated concentrations are negative and are treated as “not detected.”

### North Sea/sampling sites NS 1 (lower Saxony Wadden Sea) and NS 2 (Schleswig-Holstein Wadden Sea)—FAO/ICES division 27.4.b

#### D9 compliance assessment

**Fig. 2:**
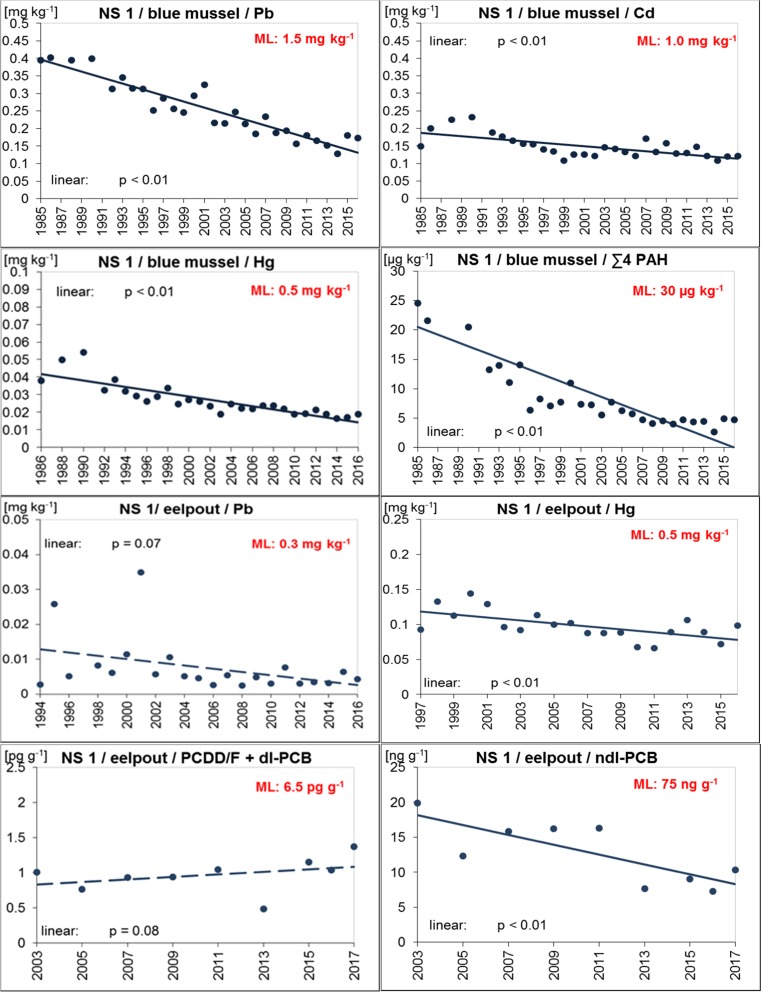
Trends of D9 relevant contaminants in ESB samples of blue mussels (soft body) and eelpout (fillet) from the North Sea/Lower Saxony Wadden Sea (coastal region of FAO/ICES Division 27.4.b). The lines represent the results of the linear regression (solid for significant linear trend, dashed for not significant). ML maximum level allowed in fish and fishery products (Reg. (EC) No. 1881/2006 and amendments; EC 2006a, 2008c, 2011a, b, c)

**Fig. 3:**
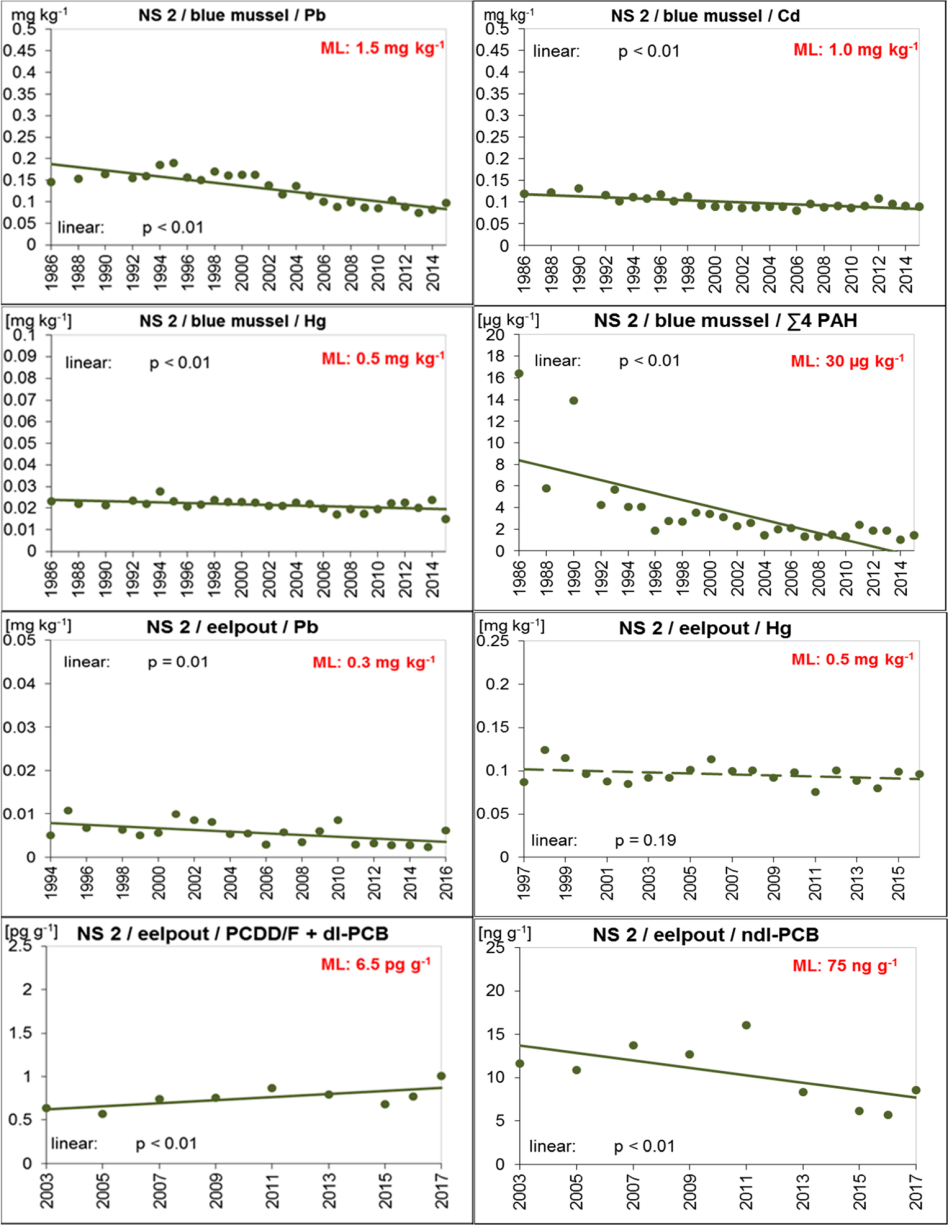
Trends of D9 relevant contaminants in ESB samples of blue mussels (soft body) and eelpout (fillet) from the North Sea/Schleswig-Holstein Wadden Sea (coastal region of FAO/ICES Division 27.4.b). The lines represent the results of the linear regression (solid for significant linear trend, dashed for not significant). ML maximum level allowed in fish and fishery products (Reg. (EC) No. 1881/2006 and amendments; EC 2006a, 2008c, 2011a, b, c))

Blue mussels from both ESB North Sea sites had Pb, Cd, Hg, ∑4 PAH, and B[a]P levels well below the maximum allowed concentrations in fishery products laid down in Reg. (EC) No. 1881/2006 (amended by Reg. (EC) No. 629/2008, Reg. (EU) No. 1259/2011, Reg. (EU) No. 420/2011, and Reg. (EU) No. 835/2011; EC 2006a, 2008c, 2011a, b, c) (Tables 2, and 3, Figs. 2, and 3). This was still true when the dilution effect caused by breathing water was considered (i.e., multiplying the wet weight concentrations by 3 at NS 1, and 2.4 at NS 2) with the notable exception of ∑4 PAHs in the early assessment years (1985–1995 and 2000 at NS 1, and 1986 and 1990 at NS 2).

Comparing both North Sea sites reveals that concentrations in mussels were always higher at sampling site NS 1 in the Lower Saxony Wadden Sea (Mann-Whitney *U* test, at least *p* = 0.02) (Tables 2 and 3, Fig. S1, Supplementary material).

Comparative data are available only for NS 1: The Lower Saxony State Office for Consumer Protection and Food Safety report mean metal concentrations of 0.23 mg kg^−1^ Pb, 0.11 mg kg^−1^ Cd, and 0.033 mg kg^−1^ Hg in blue mussels form the Lower Saxony Wadden Sea in 2016 (LAVES 2017; BMUB 2018a).

The Pb and Cd data fit well to the measured ESB mussel data from 2016 (i.e., 0.17 mg kg^−1^ Pb and 0.12 mg kg^−1^ Cd), whereas the Hg concentrations in ESB mussels from NS 1 were lower (i.e., 0.002 mg kg^−1^). When considering the dilution effect of breathing water, mussels at NS 1 had higher Pb and Cd burdens compared to the LAVES mussels (i.e., 0.52 mg kg^−1^ Pb and 0.37 mg kg^−1^ Cd), whereas Hg is still lower (0.006 mg kg^−1^).

In eelpout fillets, levels of Pb, Hg, PCDD/Fs + dl-PCBs, and ndl-PCBs were below the respective maximum levels allowed in edible fish (EC 2006a, 2008c; EC 2011a) (Figs. 2 and 3).

Since monitoring started in the mid-1990s, levels of Pb and Hg were similar in eelpout from both North Sea sites. Only in 1995 and 2001, higher Pb concentrations were observed at NS 1 (Tables 2 and 3, Figs. 2 and 3, and Fig. S2, Supplementary material).

The eelpout data are in line with the initial assessment of the German North Sea according to Article 8 of the MSFD: Concentrations of PCDD/Fs + dl-PCBs in fillet of cod (*Gadus morhua*) were below 1 ng kg^−1^ ww in 2007 (i.e., 0.380 ng kg^−1^; Karl and Lahrssen-Wiederholt 2009; BMU 2012a) and thus met the threshold value for edible fish. Hg in North Sea fish was also below the maximum allowed concentration (BMU 2012a).

#### Temporal trends

Trend analysis revealed significant decreases (*p* < 0.01) in metals and PAH contamination of blue mussels from both North Sea sites (Figs. 2 and 3). Decreases were more pronounced in mussels from NS 1 indicating that pollution has declined at this site although it was still higher compared to NS 2 in the last year of sampling (Tables 2 and 3). For PAH, strong decreases were observed at both North Sea sites where PAH levels had been high in the late 1980s and early 1990s. PAH are ubiquitous pollutants in the marine environment originating, e.g., from atmospheric deposition, offshore activities, and operational or accidental spills from ships (Brockmeyer and Theobald 2016). Possibly, the stricter regulations concerning emission and dumping of oil and oily waters that came into force in 1983 (International Convention for the Prevention of Pollution from Ships, MARPOL (1973/1978) and its amendments) resulted in the pronounced decrease of PAHs.

Cd and Hg concentrations in mussels from NS 2 decreased slightly but steadily during the monitoring period. Concentrations between the first and last year differed by only − 23% for Cd and − 19% for Hg. Even though these decreases may not have a direct environmental impact, the steadiness of the decreases nevertheless indicates that pollution is declining in the area.

For eelpout, significant decreases were detected for Hg and ndl-PCBs at NS 1 and for Pb and ndl-PCBs at NS 2 (at least *p* = 0.01). Pb has also decreased in eelpout from NS 1. This trend, however, was not significant.

In contrast, PCDD/Fs + dl-PCBs increased slightly in eelpout from both North Sea sites (significant trend, *p* = 0.01, only at NS 2) (Figs. 2 and 3). So far, we have no conclusive explanation for these increases, all the more as no respective trends are observed in sea gull eggs from nearby sites (i.e., island Mellum at NS 1 and island Trischen at NS 2) sampled by the ESB between 2008 and 2015/2016 (data not published yet).

### Baltic Sea/sampling site BS (Bodden National Park of Western Pomerania)—FAO/ICES subdivision 27.3d.24

#### D9 compliance assessment

**Fig. 4:**
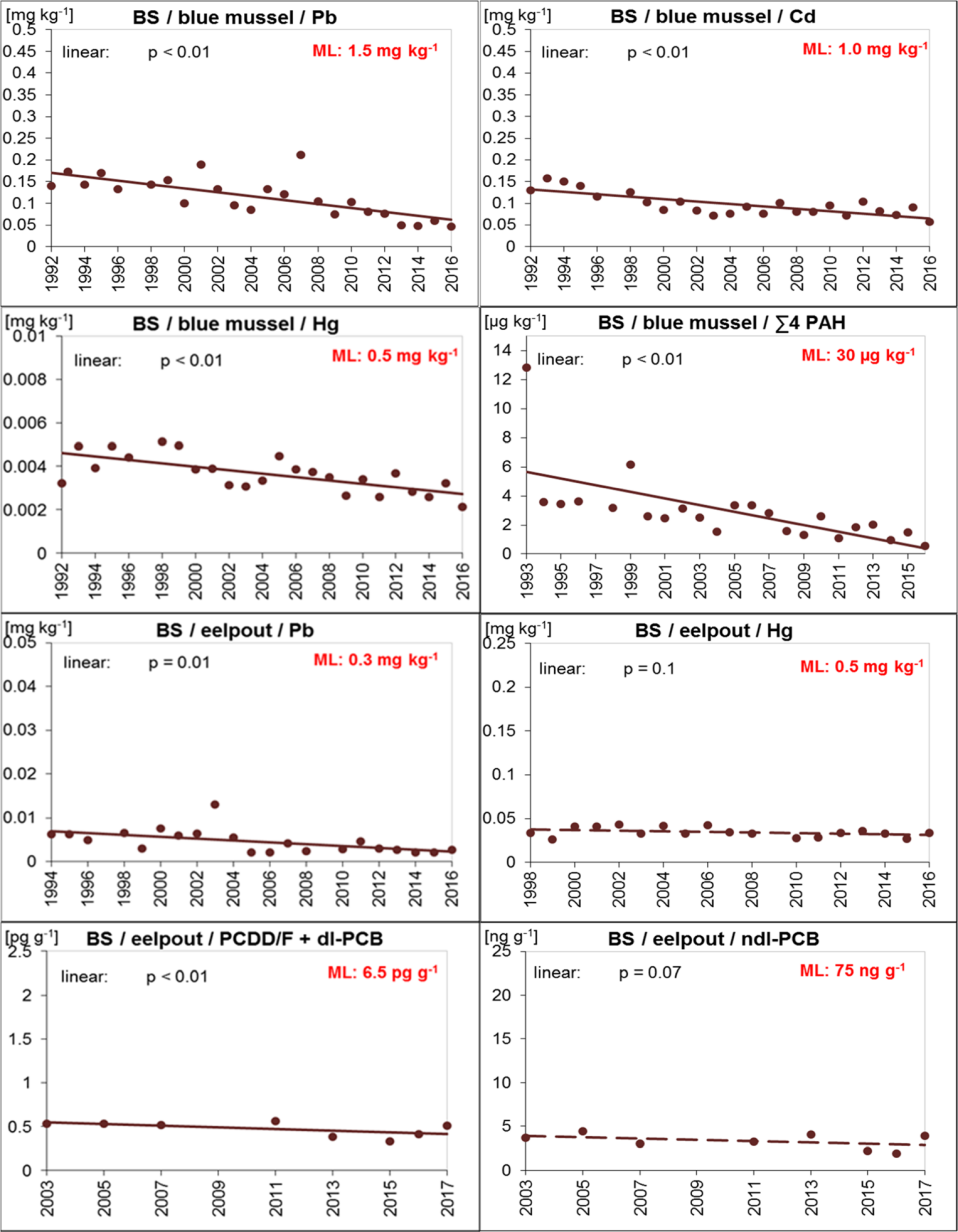
Trends of D9 relevant contaminants in ESB samples of blue mussels (soft body) and eelpout (fillet) from the Baltic Sea/Bodden National Park of Western Pomerania (coastal region of FAO/ICES Subdivision 27.3d.24). The lines represent the results of the linear regression (solid for significant linear trend, dashed for not significant). ML maximum level allowed in fish and fishery products (Reg. (EC) No. 1881/2006 and amendments; EC 2006a, 2008c, 2011a, b, c)

In mussel samples from the Baltic Sea concentrations of Pb, Cd, Hg, ∑4 PAHs, and B[a]P were well below the maximum levels allowed in fishery products as laid down in Reg. (EC) No. 1881/2006 (amended by Reg. (EC) No. 629/2008, Reg. (EU) No. 1259/2011, Reg. (EU) No. 420/2011, and Reg. (EU) No. 835/2011; EC 2006a, 2008c, 2011a, b, c) (Table 4; Fig. 4). This was, except for ∑4 PAHs in 1993, still true when considering the dilution effect caused by breathing water (i.e., multiplying the wet weight concentrations by 3).

Likewise, compliance with the threshold values was observed for eelpout fillet: During the entire monitoring period, concentrations of the D9 relevant contaminants Pb, Hg, PCDD/Fs + dl-PCBs, and ndl-PCBs in eelpout fillet were below the respective maximum levels allowed in foodstuffs (EC 2006a, 2008b, 2011a) (Table 4, Fig. 4).

These findings correspond to the initial assessment of the German Baltic Sea according to Article 8 of the MSFD: ML compliance was reported for PCDD/F + dl-PCB in fillet of cod (*Gadus morhua*) and herring (*Clupea harengus*) from the Baltic Sea (i.e., 0.228–0.631 pg g^−1^ WHO-TEQ in cod and 2.15–6.28 pg g^−1^ in herring in 2006; BMU 2012b; Karl et al. 2010) and also for ndl-PCB in herring fillets (14.7–30.4 ng g^−1^; Karl et al. 2010, ndl-PCB levels in cod were not determined). A separate study analyzed Hg in Baltic Sea fish and found no exceedance of the ML (BMU 2012b).

Similarly, the State Office for Agriculture, Food Safety and Fisheries of Mecklenburg-Western Pomerania reports Pb, Cd, Hg, and PCDD/F + dl-PCB levels in herring fillets from the Western Baltic Sea (ICES Boxes 22 and 24) sampled between 2012 to 2016 that met the respective MLs (BMUB 2018b).

#### Temporal trends

Since monitoring started, concentrations of metals and ∑4 PAHs decreased steadily in blue mussels from the ESB site in the Baltic Sea (*p* < 0.01). Again, the stricter regulation for ships (e.g., ban of rinsing of tanks and dumping the oily water into the sea, MARPOL (1973/1978) is probably responsible for the decreases. For eelpout, significant decreasing trends (*p* < 0.01) were detected for Pb, PCDD/Fs + dl-PCBs, and ndl-PCBs, whereas Hg remained more or less constant (Fig. 4).

### Evaluation of additional contaminants

Member States may choose to monitor additional contaminants not listed in Reg. (EC) No. 1881/2006 (EC 2006a, 2017a). Potentially problematic contaminants are, e.g., those listed as priority substances under the WFD (EC 2000) and assessed under Descriptor 8-C1 of the MSFD (EC 2017a). For 11 WFD priority substances, wet weight-based EQSs were derived for biota of which nine refer to fish (EC 2013b).

The biota-EQSs represent substance concentrations that are expected to be safe not only for the organisms themselves but also for human consumers (protection goal “human health”) and piscivorous predators (protection goal “secondary poisoning”; EC 2014) because the most sensitive protection goal is decisive for the EQS.

One possible trigger for inclusion in D9 assessment can be the exceedance of the biota-EQS derived for the protection of human health. An inclusion should also be considered if for any of these contaminants increasing trends are detected (Swartenbroux et al. 2010; Zampoukas et al. 2014).

In the present study, four priority substances are exemplarily analyzed that might be of relevance as additional contaminants included in D9 assessment, i.e., PBDE, HBCDD, PFOS, and TBT compounds.

The brominated flame retardants PBDE and HBCDD were widely used, e.g., in electronic products, building material, textiles, and upholstery. Technical Penta-BDE and Octa-BDE mixtures were phased out in the 1990s and are banned in the EU since 2003 (EC 2003a). The Biota-EQS of 0.0085 μg kg^−1^ fish refers to the sum of the congeners BDE-28, -47, -99, -100, -153, and -154. HBCDD was banned in 2013 (UNEP 2013) but exemptions allowed usage for building insulation materials until 2017. In the years before, it was increasingly used as substitute for PBDE. The biota-EQS for HBCDD is 167 μg kg^−1^ fish and refers to the sum of α-, β-, and γ- HBCDD.

The fluorosurfactant PFOS was used, e.g., as fabric protector and impregnation agent. Since 2008, PFOS is restricted EU-wide to only a few applications (EC 2006b). Its biota-EQS is 9.1 μg kg^−1^ fish.

TBT has long been used in antifouling paints on ships and boats. High environmental concentrations were typically associated with marinas and shipyards. In 2003, TBT was finally banned EU-wide (EC 2003b). No biota-EQS exists for TBT, but OSPAR and HELCOM have set an environmental assessment criterion (EAC) of 12 μg kg^−1^ dry weight for TBT in bivalves that relates to toxic effects on bivalves and the protection goal secondary poisoning (OSPAR 2004). Beyer et al. (2017) converted the original OSPAR EAC value to a wet weight-based EAC of 2 μg kg^−1^ ww for formulation-based TBT (using an average dry mass content for mussels of 17.38%). Blue mussels from the ESB sites had lower average dry mass contents, and accordingly, the respective wet weight-based EACs are lower (i.e., 1.13 μg kg^−1^ ww and 0.88 μg kg^−1^ ww for mussels from NS 1 and NS 2, respectively, and 0.63 μg kg^−1^ ww for mussels from BS; Tables 2, 3, and 4). Norway has derived a biota quality standard of 150 μg kg^−1^ ww for TBT compounds (NEA 2016).

**Fig. 5:**
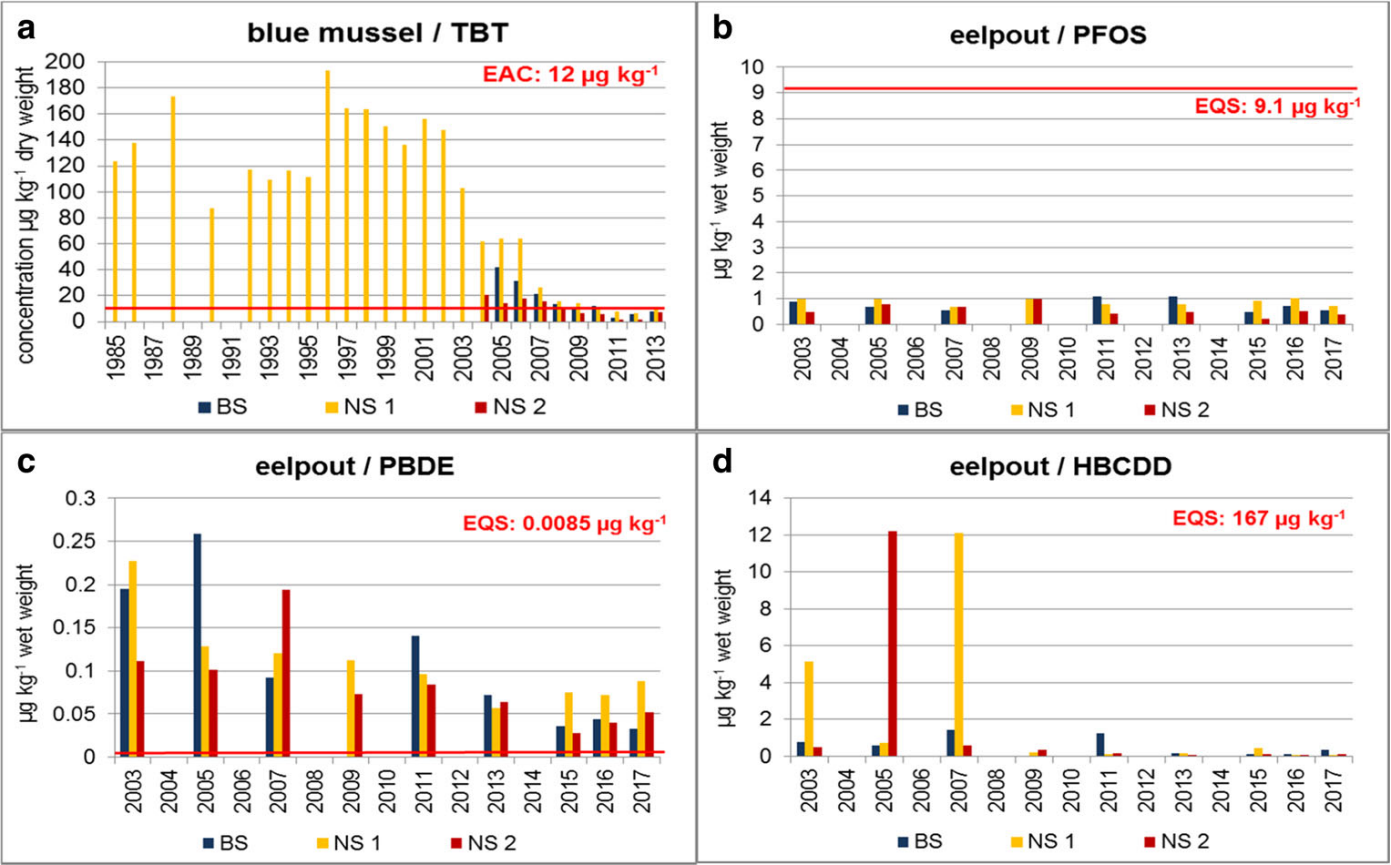
Concentrations of TBT (μg kg^−1^ dry weight, as cation) in blue mussels (soft body) (**a**) and PFOS, PBDE, and HBCDD (μg kg^−1^ wet weight) in eelpout (fillet) (**b**–**d**) from the ESB sampling sites in the North and Baltic Seas. BS Baltic Sea/Bodden National Park of Western Pomerania, NS 1 North Sea/Lower Saxony Wadden Sea, NS 2 Schleswig-Holstein Wadden Sea, EAC OSPAR environmental assessment criterion, EQS environmental quality standard

Based on retrospectively analyzed ESB samples of eelpout and blue mussel, temporal trends for TBT, PFOS, PBDE, and HBCDD have been calculated (Table S4, Fig. S4, S5, Supplementary material). Figure 5 summarizes the data from all three sampling sites.

The retrospective analysis revealed high TBT concentrations in blue mussels from the Lower Saxony Wadden Sea (NS 1) in the 1980s and 1990s that exceeded the EAC value by far (factor up to 16 based on dry weight concentrations) (Fig. 5a). Since the EU-wide ban in 2003, concentrations have declined significantly (*p* < 0.01, Table S4, Fig. S4, Supplementary material) and complied with the EAC of 12 μg kg^−1^ dw in 2010–2013. For NS 2 and BS TBT, data are available since 2004 and 2005, respectively. Lowest contamination was detected at NS 2 where mussels are not directly exposed to emissions from marinas or shipping traffic. Similar to NS 1 TBT, levels have decreased significantly in mussels from BS and NS 2 (*p* < 0.01, Table S4, Fig. S4 and S5, Supplementary material) and met the EAC in 2008 (NS 2) and 2011 (BS). Time series for TBT in mussels end in 2013 because new increases are not expected (for more details see Rüdel et al. 2003, 2009 and Radermacher 2015).

TBT levels in eelpout fillets were mostly higher than in mussels (Tables 2, 3, and 4, Fig. S3, Supplementary material). This is at least partly due to different exposure conditions as sampling sites for mussels and eelpout are not identical.

PFOS, PBDE, and HBCDD were analyzed only in eelpout because their EQSs refer to fish.

PFOS concentrations in eelpout fillet were similar at all three ESB sites and well below the respective EQS of 9.1 μg kg^−1^ (Fig. 5b). Significant changes since 2003 were only detected in fish from NS 2 (decreasing trend, *p* = 0.01; Table S4, Fig. S4, Supplementary material). For PFOS, the critical protection goal behind the EQS is human health. Accordingly, the results indicate that PFOS in marine fish from the three coastal sampling sites pose no risk to human consumers.

The picture is different for PBDE with 100% EQS exceedance at all three sites and in all years (Fig. 5c). Despite significant decreases (*p* < 0.01, Table S4, Fig. S4 and S5, Supplementary material), PBDE concentrations in eelpout fillets from all three sites still exceeded the EQS in 2016 and 2017 by factors of 4–10. The critical protection goal behind the EQS is human health implying that, despite decreasing trends, PBDE in marine fish from the three coastal sites can still pose a risk for human consumers.

HBCDD concentrations in eelpout fillet were always way below the EQS of 167 μg kg^−1^ (Fig. 5d). In most years, concentrations ranged between 0.11 and 1.5 μg kg^−1^ with higher levels detected only in 3 years (where sample handling or measurement errors cannot be excluded). Significant decreases were detected at NS 1 (*p* = 0.03) and BS (*p* < 0.01; Table S4, Fig. S4 and S5, Supplementary material). The EQS for HBCDD is based on the protection goal secondary poisoning. The quality standard derived for human health is about 36 times higher (i.e., 6100 μg kg^−1^, EC 2014). According to these data, HBCDD in marine fish from all three ESB sites pose no risk for human consumers.

## Summary and conclusions

Evaluating the suitability of the ESB samples for D9 assessment gives a mixed picture:

- The requirements of the MSFD regarding the geographical scale of D9 monitoring are only partly fulfilled: Of the catch or production area according to Art. 38 of Reg. (EU) No. 1379/2013 (EC 2013b), only a section of the German coastal regions of the Baltic Sea and the North Sea are covered. The EEZ of the Greater North Sea and the Baltic Sea are not covered at all. This is a clear limitation with respect to D9 monitoring.
  Even though the comparison of the ESB data with fish data from the open seas (Karl and Lahrssen-Wiederholt 2009; Karl et al. 2010) suggests that contamination is comparable, there are many factors that can lead to differences. Coastal areas are mainly subject to pollution originating from land, like inputs from rivers, run-off from agricultural sites, and wastewaters from industrial and municipal wastewater treatment plants, while ship traffic, dumping, off-shore activities, and atmospheric deposition are the major sources of pollution in the open sea (Davis 1993). Differences pollution-wise between coastal regions and the open sea can be further enhanced by processes like dilution, degradation, and transformation of contaminants.

- The marine specimens archived by the ESB, i.e., blue mussel soft bodies and eelpout fillets, are considered suitable for D9 monitoring although with limitations in the case of eelpout as it is not one of the most consumed fish in Germany. It has to be emphasized, however, that two species can only give an indication of the contamination of fishery products and may be representative for some of the most consumed species but by far not for all. It is well-known that fish contamination depends on biological factors like fish age, size, gender, migration behavior, trophic position, and lipid levels (e.g., Brázová et al. 2012; Burger et al. 2001; Dušek et al. 2005; Gewurtz et al. 2011; McIntyre and Beauchamp 2007; Pulkrabová et al. 2007).

- Sampling and processing of the ESB meet the requirements of the MSFD with the limitation that the personnel involved in sampling is not authorized according to Reg. (EC) No. 333/2007 and Reg. (EC) No. 644/2017 and no controls according to Reg. (EU) No 882/2004 are performed during sampling and processing.

- The requirements of the MSFD regarding analytical methods are mainly fulfilled. An exemption is the PAH analyses where the ESB-analytical method does not distinguish between benzo[b]fluoranthene, benzo[j]fluoranthene, and benzo[k]fluoranthene and between chrysene and triphenylene.

Taken together, it can be concluded that the ESB samples of blue mussels and eelpout fillet are basically suitable for D9 assessment but should be complemented by additional georeferenced samples of other species also from the EEZ.

As long as such samples are not available, an indicative assessment of D9 could be based on ESB samples.

The big advantage of the ESB samples is their clear assignment to a specific marine region. The samples can be used for D8-C1 and D9 assessment which can help to save costs. Furthermore, the archived samples of the ESB offer the opportunity of retrospective analysis to assess time series also of contaminants that may be included in D9 assessment in the future.

The limitations regarding official controls and authorized personnel should be noted, but it should also be considered that the ESB works according to strict standard operating procedures and the whole process from sampling to chemical analyses follows the EN ISO/IEC 17025 standard.

The deviation between the MSFD requirements and the ESB data regarding ∑4 PAHs are not viewed as limitation but rather as a worst-case scenario because additional substances are included (i.e., if the ESB data comply with the ML for ∑4 PAHs, this will also be true when considering only the four required PAHs). Nevertheless, in the future, a more selective method for PAHs could be applied to analyze benzo[b]fluoranthene and chrysene.

The exemplary D9 assessment at the ESB sites showed that 100% of the blue mussel and eelpout samples from the North Sea (coastal areas of FAO/ICES Division 27.4.b) and the Baltic Sea (coastal area of FAO/ICES Subdivision 27.3d.24) sampled since the 1990s met the threshold values for Pb, Cd, Hg, ∑4 PAHs, PCDD/Fs + dl-PCBs, and ndl-PCBs in foodstuffs listed in Regulation (EC) No. 1881/2006 and its amendments (EC 2006a, 2008c, 2011a, b, c). When considering the dilution caused by breathing water in blue mussels, the thresholds were met at the latest since 2001. The additionally assessed contaminants PFOS and HBCDD were below the respective WFD biota-EQS in eelpout fillets and are considered as safe for human consumers. In contrast, PBDE levels in eelpout fillet exceeded the respective EQS indicating that a risk to human consumers cannot be excluded. TBT analysis ended in 2013 after concentrations in blue mussels had met the EAC for at least three consecutive years.

## Electronic supplementary material

ESM 1 (DOCX 566 kb)
