# Supplementary material for: Assessment of seafood contamination under the marine strategy framework directive: contributions of the German environmental specimen bank
Source: Environ Sci Pollut Res Int. 2018 Jul 14;25(27):26939–56. doi: 10.1007/s11356-018-2728-1 (PMC6132394; doi:10.1007/s11356-018-2728-1)
Supplement: Supplementary file 1 — (DOCX 566 kb) [file 11356_2018_2728_MOESM1_ESM.docx]

**Assessment of seafood contamination under the Marine Strategy Framework Directive: Contributions of the German Environmental Specimen Bank**

Annette Fliedner^1^, Heinz Rüdel^1^, Burkhard Knopf^1^, Nina Lohmann^2^, Martin Paulus^3^, Michael Jud^4^, Ulrike Pirntke^5^ Jan Koschorreck^5^

^1^ Fraunhofer Institute for Molecular Biology and Applied Ecology (Fraunhofer IME), Department Environmental Specimen Bank and Elemental Analysis, 57392 Schmallenberg, Germany

^2^ Eurofins GfA Lab Service GmbH, Neulaender Kamp 1a, 21079 Hamburg, Germany

^3^ Department of Biogeography / Environmental Specimen Bank, Trier University, 54286 Trier, Germany

^4^ Federal Office of Consumer Protection and Food Safety (BVL), Unit 101, P.O. Box 110260, 10832 Berlin, Germany

^5^ German Environment Agency (Umweltbundesamt), 06813 Dessau-Rosslau, Germany

**Supplementary material**

**Table S1**: Comparison of food legislation requirements and ESB standards regarding sampling and processing (BVL 2017; EC 2017)

|  | **Food legislation requirements**  Reg. (EU) No 644/2017; Reg. (EC) No 333/2007 and amendments; BVL (2013, 2017) | **ESB procedures**  (Standard operating procedures; [www.umweltprobenbank.de](http://www.umweltprobenbank.de)); accredited following the EN ISO/IEC 17025 standard |
| --- | --- | --- |
| **Sampling site** |  | - Representative for ecosystem - Sufficient size and population density - not in direct neighborhood of local emission sources |
| **Documentation** |  | Detailed sampling protocol incl. biometric parameters |
| **Sampling** | Fish:   - authorized personnel - temporal and geographical scope of sampling shall provide a representative sample of the specified contaminants in seafood in the marine region or subregion. - fish are considered as being of comparable size and weight in case the difference in size and weight does not exceed about  50 %.   Mussels:   - authorized personnel - temporal and geographical scope of sampling shall provide a representative sample of the specified contaminants in seafood in the marine region or subregion. - at least 1 kg whole mussel incl. shell. | Eelpout:   - professional fishermen / trained personnel, authorized by the Federal Environment Agency - sampling in May/June prior to mating season - 20 – 200 individual fish of all available size classes - keeping of living fish in habitat water until processing   Blue mussels:   - trained personnel, authorized by the Federal Environment Agency - sampling: North Sea: every second month starting in February;  Baltic Sea: twice per year (June and November) - ≥ 300 individual mussels per year, largest size at sampling site - mussels are deep frozen on site |
| **Sample preparation** | Blue mussel:   - opened and placed on tissue to eliminate water inside the shells - soft bodies are carefully removed and pooled   Fish (incl. deep frozen):   - ≥ 0.5 – 1 kg skinless fillets (whole fish or middle sections depending on fish size) - aggregate samples of ≥ 1 kg are generated uniting incremental samples (each of which is ≥ 100 g). (Maximum Level applies to muscle meat without skin or, when whole fish is eaten, to whole fish). - samples are homogenized and either analyzed immediately or deep frozen | Blue mussel:   - soft bodies are separated from shell while maintaining the cold chain - respiratory water and intestinal content remain in the mussel as part of the sample - annual pool samples of 1.1 kg soft bodies   Eelpout:   - 1.1 kg skinless fillet (corresponding to 20 – 200 individual fish of all available size classes). - fillets are deep frozen on site - samples are homogenized while maintaining the cold chain and annual pool samples are prepared |
| **Laboratories** | - accredited following the EN ISO/IEC 17025 standard - successful participation in interlaboratory studies - participate in proficiency testing schemes (IUPAC/ISO/AOAC) - internal quality control procedure (ISO/ AOAC/IUPAC) | - accredited following the EN ISO/IEC 17025 standard - successful participation in interlaboratory studies - participate in proficiency testing schemes (Quasimeme, [www.quasimeme.org](http://www.quasimeme.org); Norwegian Institute of Public Health, [www.fhi.no](http://www.fhi.no)) - internal quality control procedure (EN ISO/IEC 17025) |

**Table S2 (a): Performance criteria for methods of analysis for lead, cadmium and mercury referring to foods specified in Regulation (EC) No 1881/2006**red shading: Requirements according to Reg. (EC) No. 333/2007 (EC 2007), amended by Reg. (EU) 836/2011 and Reg. (EU) No. 582/2016 (EC 2011; EC 2016); white fields: ESB.
Concentration data refer to wet weight. Abbreviations see below

| **Lead (Pb)  Parameter** | **Criterion** | | | | |
| --- | --- | --- | --- | --- | --- |
| **Specificity** | Free from matrix or spectral interferences | | | | |
|  | The applied ICP-MS method is free from matrix or spectral interferences | | | | |
| **Repeatability  (RSD r)** | HORRAT_r_ less than 2 | | | | |
|  | Measured RSD_r_ < 5 - 10 % predicted PRSD_r_ for typical Pb concentrations in mussels of 0.1 mg kg^-1^ =  10^-7^ kg kg^-1^: 22 % (= 2 C^–0.15^)  RSD_r_/PRSD_r_ = 0.2 - 0.5; requirement fulfilled since < 2 | | | | |
| **Reproducibility  (RSD R)** | HORRAT_R_ less than 2 | | | | |
|  | Measured RSD_R_ = 10 % (from Quasimeme proficiency test) predicted PRSD_r_ for typical Pb concentrations in mussels of 0.2 mg kg^-1^ =  2 * 10^-7^ kg kg^-1^: 20 % (= 2 C^–0.15^)  RSD_r_/PRSD_r_ = 0.5; requirement fulfilled since < 2 | | | | |
| **Recovery^1^** |  | | | | |
|  | Recovery > 85 % as determined by measurements of certified reference materials measured routinely along with each set of samples | | | | |
| **LOD^2^** | Three tenth of LOQ | | | | |
|  | 0.00006 mg kg^-1^ | | | | |
| **LOQ^2^** | Lead  (ML: 0.3 mg kg^-1^ fish,  1.5 mg kg^-1^ mussels) | ML ≤ 0.01 mg kg^-1^ | 0.01 < ML ≤ 0.02 mg kg^-1^ | 0.02 < ML < 0.1 mg kg^-1^ | ML ≥ 0.1 mg kg^-1^ |
|  |  | ≤ ML | ≤ two thirds of the ML | ≤ two fifths of the ML | ≤ one fifth of the ML = 0.06 mg kg^-1^ |
|  |  | - | - | - | 0.0002 mg kg^-1^ |

| **Cadmium (Cd)  Parameter** | **Criterion** | | |
| --- | --- | --- | --- |
| **Specificity** | Free from matrix or spectral interferences | | |
|  | The applied ICP-MS method is free from matrix or spectral interferences | | |
| **Repeatability  (RSD r)** | HORRAT_r_ less than 2 | | |
|  | Measured RSD_r_ < 5 - 10 % predicted PRSD_r_ for typical Cd concentrations in mussels of 0.1 mg kg^-1^ =  10^-7^ kg kg^-1^: 22 % (= 2 C^–0.15^)  RSD_r_/PRSD_r_ = 0.2 - 0.4; requirement fulfilled since < 2 | | |
| **Reproducibility  (RSD R)** | HORRAT_R_ less than 2 | | |
|  | Measured RSD_R_ = 7 % (from Quasimeme proficiency test) predicted PRSD_r_ for typical Cd concentrations in mussels of 0.2 mg kg^-1^ =  2 * 10^-7^ kg kg^-1^: 20 % (= 2 C^–0.15^)  RSD_r_/PRSD_r_ = 0.4; requirement fulfilled since < 2 | | |
| **Recovery^1^** |  | | |
|  | Recovery > 95 % as determined by measurements of certified reference materials measured routinely along with each set of samples | | |
| **LOD^2^** | Three tenth of LOQ | | |
|  | 0.00002 mg kg^-1^ | | |
| **LOQ^2^** | Cadmium  (ML: 1.0 mg kg^-1^ mussels) | ML < 0.100 mg kg^-1^ | ML ≥ 0.100 mg kg^-1^ |
|  |  | ≤ two fifth of the ML | ≤ one fifth of the ML = 0.2 mg kg^-1^ |
|  |  | - | 0.00005 mg kg^-1^ |

| **Mercury (Hg)  Parameter** | **Criterion** | | |
| --- | --- | --- | --- |
| **Specificity** | Free from matrix or spectral interferences | | |
|  | The applied AAS method is free from matrix or spectral interferences | | |
| **Repeatability  (RSD r)** | HORRAT_r_ less than 2 | | |
|  | Measured RSD_r_ < 5 % predicted PRSD_r_ for typical Hg concentrations in fish of 0.04 mg kg^-1^ =  4 * 10^-8^ kg kg^-1^: 26 % (= 2 C^–0.15^)  RSD_r_/PRSD_r_ = 0.2; requirement fulfilled since < 2 | | |
| **Reproducibility  (RSD R)** | HORRAT_R_ less than 2 | | |
|  | Measured RSD_R_ = 19 % (from Quasimeme proficiency test) predicted PRSD_r_ for typical Hg concentrations in mussels of 0.1 mg kg^-1^ =  10^-7^ kg kg^-1^: 22 % (= 2 C^–0.15^)  RSD_r_/PRSD_r_ = 0.9; requirement fulfilled since < 2 | | |
| **Recovery^1^** |  | | |
|  | Recovery range 90 -110 % as determined by measurements of certified reference materials measured routinely along with each set of samples | | |
| **LOD^2^** | Three tenth of LOQ | | |
|  | 0.00002 mg kg^-1^ | | |
| **LOQ^2^** | Mercury (ML:  0.5 mg kg^-1^ fish) | ML < 0.100 mg kg^-1^ | ML ≥ 0.100 mg kg^-1^ |
|  |  | ≤ two fifth of the ML | ≤ one fifth of the ML = 0.1 mg kg^-1^ |
|  |  | - | 0.00008 mg kg^-1^ |

^1^ Recovery calculations (Reg (EC) No. 333/2007): If an extraction step is applied in the analytical method, the analytical result shall be corrected for recovery. In this case the level of recovery must be reported. In case no extraction step is applied in the analytical method (e.g., in case of metals), the result may be reported uncorrected for recovery if evidence is provided by ideally making use of suitable certified reference material that the certified concentration allowing for the measurement uncertainty is achieved (i.e., high accuracy of the measurement), and thus that the method is not biased. In case the result is reported uncorrected for recovery this shall be mentioned.
^2^ The LOQ was estimated using blank value measurements (estimation of the LOD using the blank value method according to the German standard DIN 32645, the LOQ being calculated as three times the LOD);
ML: Maximum level allowed in foodstuffs. HORRAT: see Horwitz et al. 1980, Horwitz and Albert 2006, Thomson 2000; RSD: relative standard deviation in %; PRSD: Horwitz-Predicted RSD.

**Table S2 (b): Performance criteria for methods of analysis for** **polycyclic aromatic hydrocarbons** **referring to foods specified in Regulation (EC) No 1881/2006**
red shading: Requirements according to Reg. (EC) No. 333/2007 (EC 2007), amended by Reg. (EU) No. 836/2011 and Reg. (EU) No. 582/2016 (EC 2011d; EC 2016); white fields: ESB

| **Parameter** | **Criterion** | | | | | | | | | |
| --- | --- | --- | --- | --- | --- | --- | --- | --- | --- | --- |
| **Specificity** | Free from matrix or spectral interferences, verification of positive detection | | | | | | | | | |
| **Repeatability (RSD r )** | HORRAT r less than 2 | | | | | | | | | |
|  |  | | B[a]P | | B[a]A | | B[b,j,k]F | | C+T | Comment |
|  | Measured RSD_r_ (%) | | 16 | | 14 | | 14 | | 14 | Regular analysis of ESB-blue mussel |
|  | Typical concentration: | (µg kg^-1^) | 1 | | 1 | | 5 | | 3 |  |
|  |  | (kg kg_-1_) | 1 * 10^-9^ | | 1 * 10^-9^ | | 5 * 10^-9^ | | 3 * 10^-9^ |  |
|  | Predicted PRSD_r_ for typical concentration (%) | | 45 | | 45 | | 35 | | 38 | (= 2 c^-0,15^) |
|  | RSD_r_/PRSD_r_ | | 0.4 | | 0.3 | | 0.4 | | 0.4 |  |
|  | Fulfilment of requirement: | | yes | | yes | | yes | | yes | since RSD_r_/PRSD_r_ < 2 |
| **Reproducibility (RSD R )** | HORRAT R less than 2 | | | | | | | | | |
|  |  | | B[a]P | | B[a]A | | B[b]F | | C+T | Comment |
|  | Measured RSD_R_ (%) | | 18 | | 16 | | 16 | | 19 | Quasimeme |
|  | Typical concentration: | (µg kg^-1^) | 3 | | 3 | | 3 | | 3 |  |
|  |  | (kg kg^-1^) | 3 * 10^-9^ | | 3 * 10^-9^ | | 3 * 10^-9^ | | 3 * 10^-9^ |  |
|  | Predicted PRSD_R_ for typical concentration (%) | | 39 | | 38 | | 37 | | 38 | (= 2 c^-0,15^) |
|  | RSD_R_/PRSD_R_ | | 0.5 | | 0.5 | | 0.4 | | 0.5 |  |
|  | Fulfilment of requirement: | | yes | | yes | | yes | | yes | since RSD_R_/PRSD_R_ < 2 |
| **Recovery** | 50 – 120 % | | | | | | | | | |
|  |  | | | | | | | | | |
| **LOD** | ≤ 0.30 µg kg^-1^ for each of the four substances | | | | | | | | | |
|  |  | B[a]P | | B[a]A | | B[b,j,k]F | | C+T | | Comment |
|  | LOQ (µg kg^-1^) | 0.03 – 0.04 | | 0.05 – 0.06 | | 0.1 – 0.2 | | 0.2 – 0.3 | |  |
|  | Fulfilment of requirement: | yes | | yes | | yes | | yes | | since ≤ 0.30 µg kg^-1^ |
| **LOQ** | ≤ 0.90 µg kg^-1^ for each of the four substances | | | | | | | | | |
|  |  | B[a]P | | B[a]A | | B[b,j,k]F | | C+T | |  |
|  | LOQ (µg kg^-1^) | 0.06 – 0.08 | | 0.05 – 0.06 | | 0.3 – 0.4 | | 0.4 – 0.5 | |  |
|  | Fulfilment of requirement: | yes | | yes | | yes | | yes | | since ≤ 0.90 µg kg^-1^ |

HORRAT r = The observed RSD r divided by the PRSD r value estimated from the (modified) Horwitz equation (Thompson, 2000) using the assumption r = 0.66 R.
HORRAT R ’ = The observed RSD R divided by the PRSD R value estimated from the (modified) Horwitz equation (Thompson 2000).
B[a]P = Benzo[a]pyrene, B[a]A = Benz[a]anthracene, B[b,j,k]F = Benzo[b,j,k]fluoranthene,
B[b]F = Benzo[b]fluoranthene, C+T = Chrysene / Triphenylene

**Table S2 (c): Performance criteria for methods of analysis for PCDD/Fs + dl-PCBs
(Reg. (EU) 2017/644)**red shading: Requirements according to Reg. (EU) 2017/644 (EC 2017); white fields: ESB

| **Sum of dioxins and DL-PCBs as WHO(2005)-PCDD/F+PCB-TEQ (upperbound)**  **Parameter** | **requirements** | |
| --- | --- | --- |
|  | **Screening with bioanalytical or physico- chemical methods^1^** | **Confirmatory methods^2^** |
| LOQ |  | One fifth of ML |
|  |  | Typically well below 1/25 of ML |
| False-compliant rate^3^ | < 5% |  |
|  | --- |  |
| Trueness |  | -20 to + 20% |
|  |  | -13 % (reference material EDF-2525) |
| Repeatability (RSD_r_) | < 20% |  |
|  | --- |  |
| Intermediate precision (RSD_R_) | < 25% | < 15% |
|  | --- | 14 % |
| Difference between upper- and lowerbound level ^3^ |  | ≤ 20 % |
|  |  | fulfilled^4^ |

^1^bioanalytical or GC-MS methods; ^2^GC-HRMS or GC-MS/MS. ^3^With respect to maximum levels (ML);
^4^Requirement is checked on a per sample basis. Only data sets will be regarded as valid fulfilling this requirement.

**Table S2 (d): Performance characteristics / Criteria for the sum of the six indicator ndl-PCBs at the maximum level (Reg. (EU) 2017/644)**

red shading: Requirements according to Reg. (EU) 2017/644 (EC 2017); white fields: ESB

| **Parameter** | **Isotope dilution mass spectrometry** | **Other techniques** |
| --- | --- | --- |
| LOQ | ≤ one third of ML | |
|  | Typically well below 1/100 of ML | --- |
| Trueness | -20 to +20% | -30 to +30% |
|  | +9% (reference material EDF-2525) | --- |
| Intermediate precision (RSD%) | ≤ 15% | ≤ 20% |
|  | 11% | --- |
| Difference between upper and lower bound calculation | ≤ 20% | ≤ 20% |
|  | fulfilled^1^ | --- |
| Recovery | 60 – 120 %^2^ | 60 – 120 % |
|  | fulfilled^1^ | --- |

^1^Requirement is checked on a per sample basis. Only data sets will be regarded as valid that fulfil this requirement; ^2^Lower or higher recoveries for individual congeners with a contribution to the sum of non-dioxin-like PCBs below 10 % are acceptable, when all six isotope-labelled non-dioxin-like PCB congeners are used for quantification

**Table S2 (e): Performance criteria for methods of analysis for PBDE, HBCDD, and PFOS in fish and seafood (Swartenbroux et al. 2010; Webster et al. 2010 a+b, EC 2014)**

| **contaminant** | **parameter** | **MSFD requirements** | **ESB** |
| --- | --- | --- | --- |
| PBDE (BDE-28, -47, -99, -100, -153, -154) | LOQ | 0.01 µg kg^-1^ ww | 0.005 – 0.007 µg kg^-1^ ww |
|  | Recovery: | 70 – 120% | Formal acceptance criterion: 50 – 120%^1^  Typical for eelpout: 70 – 120% |
| HBCDD  (α-, β-, γ-diastereomers) | LOQ | 0.01 µg kg^-1^ ww | 0.003 – 0.005 µg kg^-1^ ww |
|  | Recovery | 70 – 120% | 50 – 120%^1^ |
| PFOS | LOQ | 1 µg kg^-1^ | 0.05 – 0.07 µg kg^-1^ ww |
|  | Recovery | 70 – 120% | 50 – 120%^1^ |

^1^Requirement is checked on a per sample basis. Only data sets will be regarded as valid that fulfil this requirement.

**Table S3: Available ESB data used to assessDescriptor 9 of the MSFD.** Red shading: substances that may be relevant in future assessments.
Data refer to coastal sampling sites in the Baltic West of Bornholm (FAO/ICES Subdivision 27.3d.24) and the Central North Sea (FAO/ICES Division 27.4.b).
n: number of annual pool samples; BS: Baltic Sea / Bodden National Park of Western Pommerania; NS 1: North Sea / National Park Biosphere Reserve Lower Saxony Wadden Sea; NS 2: North Sea / National Park Biosphere Reserve Schleswig-Holstein Wadden Sea

| **Contaminant** | **Sampling site** | **Eelpout (fillet)** | | **Blue mussel  (soft body)** | |
| --- | --- | --- | --- | --- | --- |
|  |  | n | period | n | period |
| **Lead** | BS | 21 | 1998 - 2016 | 24 | 1992 - 2016 |
|  | NS1 | 22 | 1994 - 2016 | 29 | 1985 - 2016 |
|  | NS2 | 22 | 1994 - 2016 | 27 | 1986 - 2015 |
| **Cadmium** | BS |  |  | 24 | 1992 - 2016 |
|  | NS1 |  |  | 29 | 1985 - 2016 |
|  | NS2 |  |  | 27 | 1986 - 2015 |
| **Mercury** | BS | 18 | 1998 - 2016 | 24 | 1992 - 2016 |
|  | NS1 | 20 | 1997 - 2016 | 28 | 1986 - 2016 |
|  | NS2 | 20 | 1997 - 2016 | 27 | 1986 - 2015 |
| **PCDD/Fs+dl-PCBs** | BS | 8 | 2003 – 2017 |  |  |
|  | NS1 | 9 | 2003 – 2017 |  |  |
|  | NS2 | 9 | 2003 - 2017 |  |  |
| **6 ICES ndl-PCBs** | BS | 8 | 2003 - 2017 |  |  |
|  | NS1 | 9 | 2003 - 2017 |  |  |
|  | NS2 | 9 | 2003 - 2017 |  |  |
| **∑PAH** | BS |  |  | 23 | 1993 - 2016 |
|  | NS1 |  |  | 29 | 1985 - 2016 |
|  | NS2 |  |  | 27 | 1986 - 2015 |
|  |  |  |  |  |  |
| **TBT** | BS | 16 | 1994 - 2013 | 9 | 2005 - 2013 |
|  | NS1 | 17 | 1994 - 2013 | 25 | 1986 - 2013 |
|  | NS2 | 12 | 1999 - 2013 | 10 | 2004 - 2013 |
| **PFOS** | BS | 8 | 2003 – 2017 |  |  |
|  | NS1 | 9 | 2003 - 2017 |  |  |
|  | NS2 | 9 | 2003 - 2017 |  |  |
| **PBDE** | BS | 8 | 2003 - 2017 |  |  |
|  | NS1 | 9 | 2003 - 2017 |  |  |
|  | NS2 | 9 | 2003 - 2017 |  |  |
| **HBCDD** | BS | 8 | 2003 - 2017 |  |  |
|  | NS1 | 9 | 2003 - 2017 |  |  |
|  | NS2 | 9 | 2003 - 2017 |  |  |

n: total number of annual pool samples.

**Table S4:** Trend data of TBT in blue mussel (soft body) and TBT, PFOS, PBDE and HBCDD in eelpout fillet from the North Sea / ESB sampling sites NS 1 (Lower Saxony Wadden Sea; FAO/ICES Division 27.4.b), NS 2 (Schleswig-Holstein Wadden Sea ; FAO/ICES Division 27.4.b) and the Baltic Sea / ESB sampling site BS (Bodden National Park of Western Pomerania; FAO/ICES Subdivision 27.3d.24)

| Specimen type | Contaminants | Sampling sites | Monitoring period | n | linear trend α = 0.05 | | Contrast first year – last year | |
| --- | --- | --- | --- | --- | --- | --- | --- | --- |
|  |  |  |  |  | Trend direction | p-value | % change | p-value |
| Blue mussel  (soft body) | TBT | NS 1 | 1986 - 2013 | 25 | decrease | < 0.01 | -97 | < 0.01 |
|  |  | NS 2 | 2004 - 2013 | 10 | decrease | < 0.01 | -75 | < 0.01 |
|  |  | BS | 2005 - 2013 | 9 | decrease | < 0.01 | -84 | < 0.01 |
|  | | | | | | | | |
| Eelpout  (fillet) | TBT | NS 1 | 1994 - 2013 | 17 | decrease | < 0.01 | -65 | 0.006 |
|  |  | NS 2 | 1999 - 2013 | 12 | *decrease* | n.s. | -22 | n.s. |
|  |  | BS | 1994 - 2013 | 16 | decrease | < 0.01 | -90 | < 0.01 |
|  | PFOS | NS 1 | 2003 - 2017 | 9 | *decrease* | n.s. | -17 | n.s. |
|  |  | NS 2 | 2003 - 2017 | 9 | decrease | 0.01 | -31 | n.s. |
|  |  | BS | 2003 - 2017 | 8 | *decrease* | n.s. | -44 | < 0.01 |
|  | PBDE | NS 1 | 2003 - 2017 | 9 | decrease | < 0.01 | -61 | < 0.01 |
|  |  | NS 2 | 2003 - 2017 | 9 | decrease | < 0.01 | -60 | 0.040 |
|  |  | BS | 2003 - 2017 | 8 | decrease | < 0.01 | -87 | < 0.01 |
|  | HBCDD | NS 1 | 2003 - 2017 | 9 | decrease | 0.032 | -97 | n.s. |
|  |  | NS 2 | 2003 - 2017 | 9 | *decrease* | n.s. | -98 | n.s. |
|  |  | BS | 2003 - 2017 | 8 | decrease | < 0.01 | -64 | 0.034 |

n: number of annual pool samples included in trend analysis; when trend direction is written in *italics*, the trend is not significant; n.s.: not significant


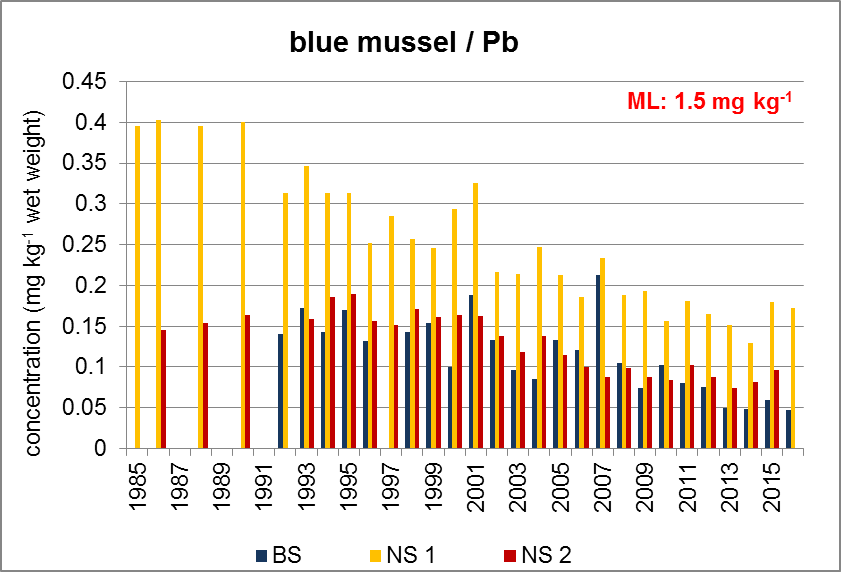

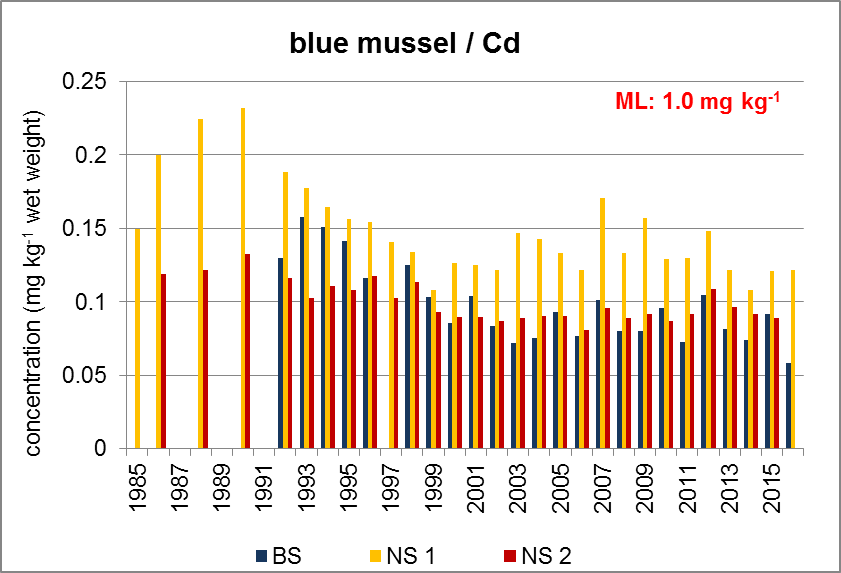

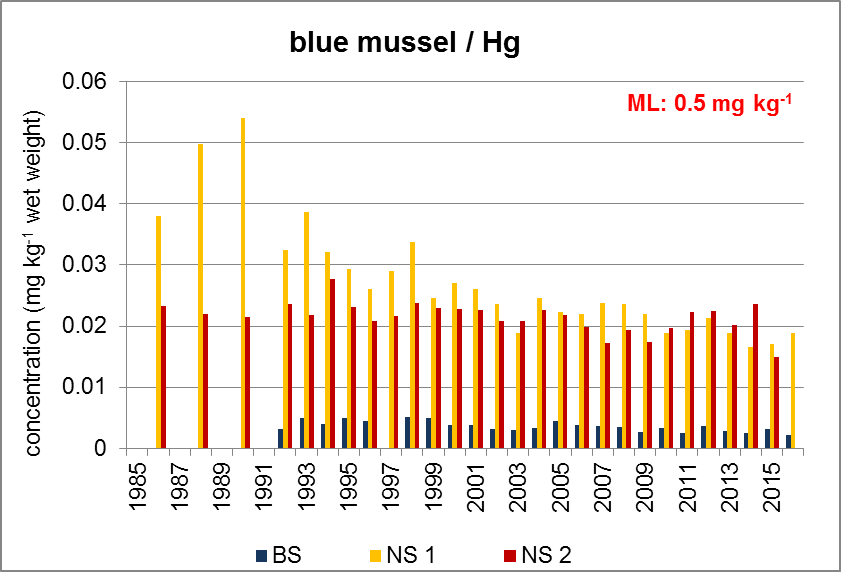

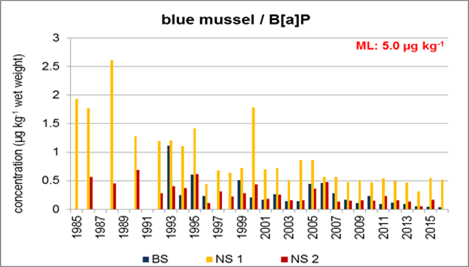

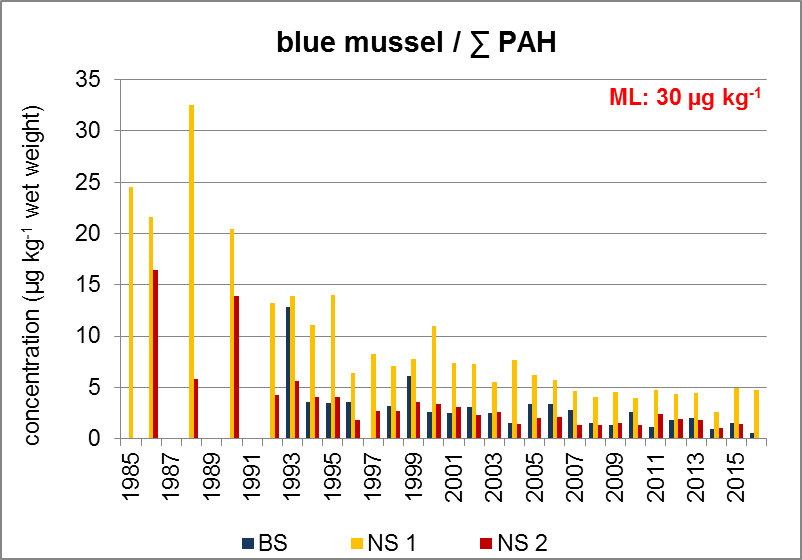


Fig. S1: Concentrations of lead (Pb), cadmium (Cd), mercury (Hg), Benzo[a]pyrene and sum of benzo[a]pyren, benzo[a]anthracene, benzo[b,j,k]fluoranthene and chrysene + triphenylene in blue mussels (*Mytilus edulis*) sampled at coastal sites in the Baltic and North Seas.
BS: Baltic Sea / Bodden National Park of Western Pomerania; NS 1: North Sea / Lower Saxony Wadden Sea; NS 2: North Sea / Schleswig-Holstein Wadden Sea; ML: Maximum level allowed in foodstuff


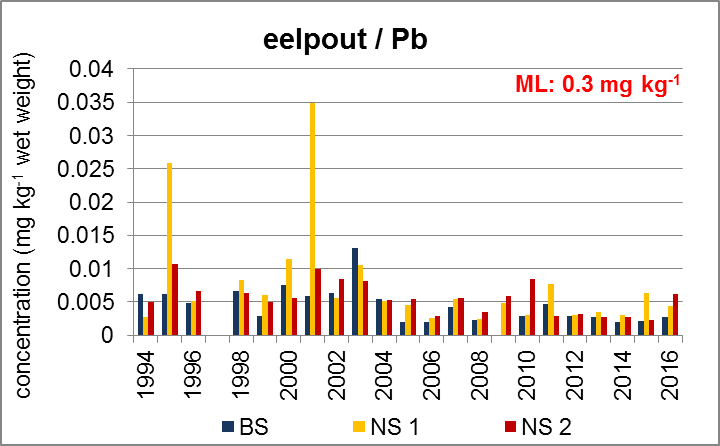

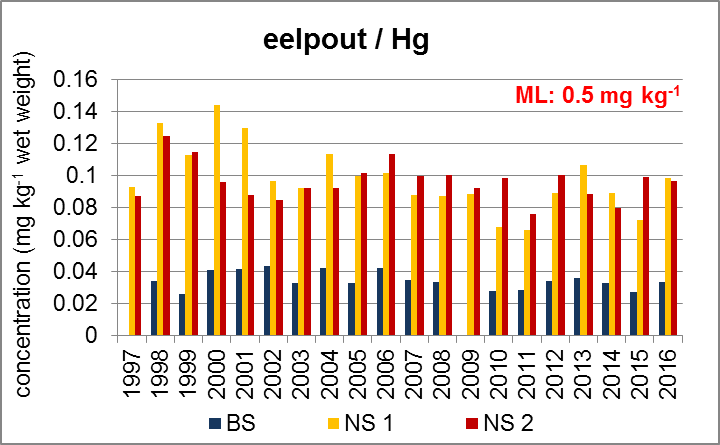

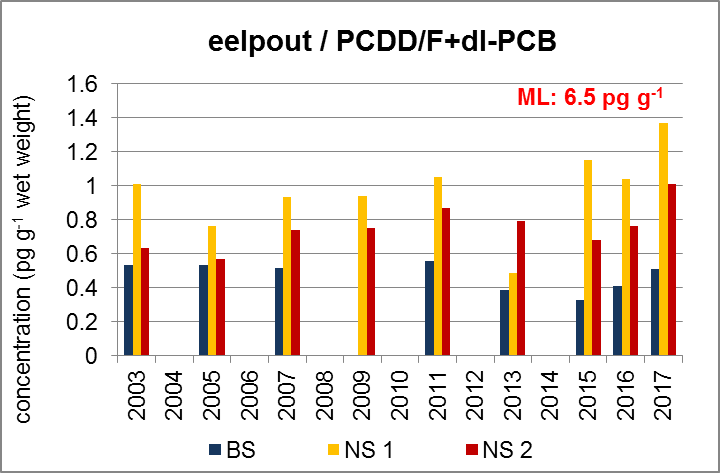

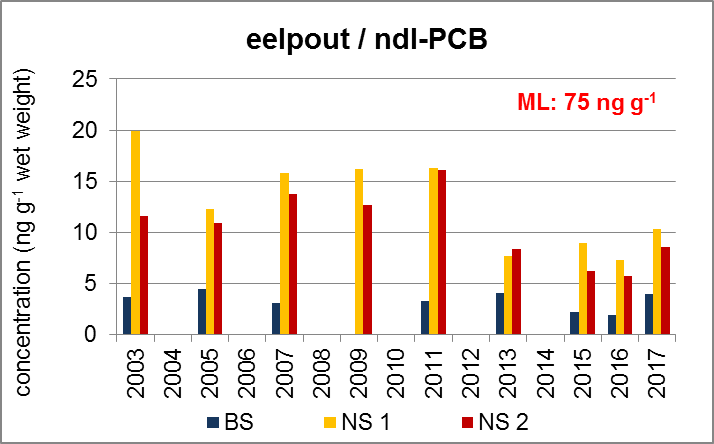


Fig. S2: Concentrations of lead (Pb), mercury (Hg), dioxins and dioxin-like PCB (PCDD/F+dl-PCB) and the sum of 6 ICES indicator-PCBs (ndl-PCB) in fillet of eelpout (*Zoarces viviparus*) sampled at coastal sites in the Baltic and North Seas.
BS: Baltic Sea / Bodden National Park of Western Pomerania; NS 1: North Sea / Lower Saxony Wadden Sea; NS 2: North Sea / Schleswig-Holstein Wadden Sea; ML: Maximum level allowed in foodstuff


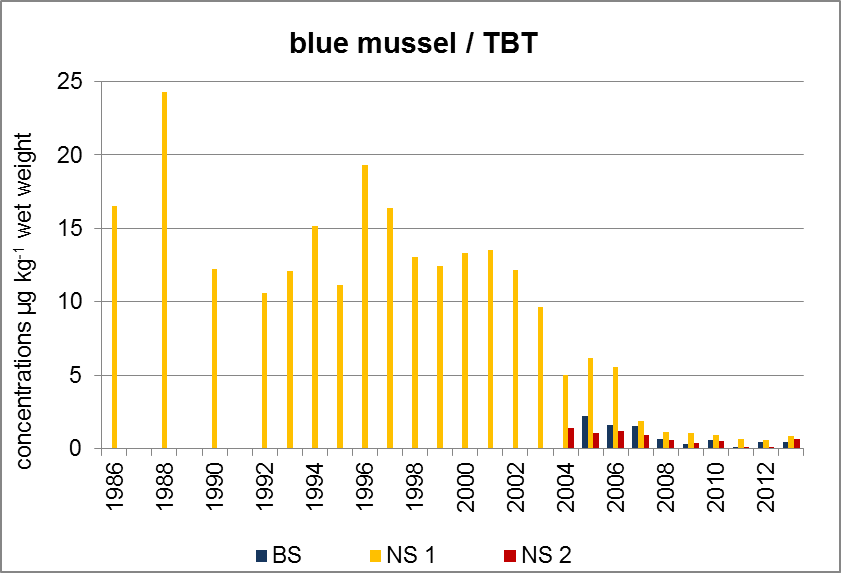

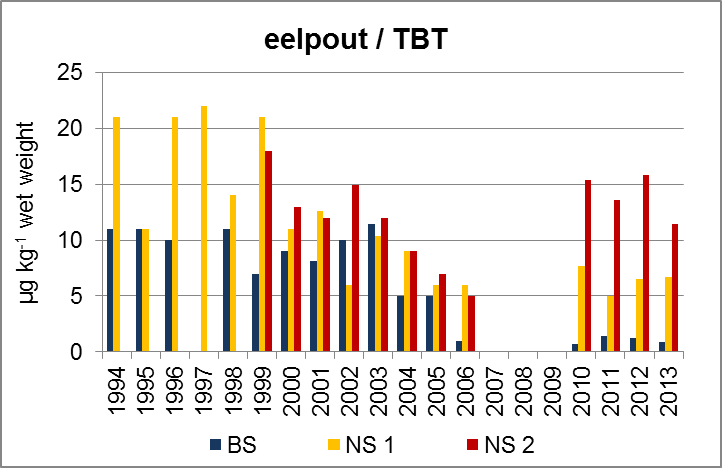


Fig. S3: Concentrations of TBT (µg kg^-1^ wet weight) in blue mussels (*Mytilus edulis*) and eelpout (*Zoarces viviparous,* fillet) from the ESB sampling sites in the North and Baltic Seas.
BS: Baltic Sea / Bodden National Park of Western Pomerania; NS 1: North Sea / Lower Saxony Wadden Sea; NS 2: North Sea / Schleswig-Holstein Wadden Sea


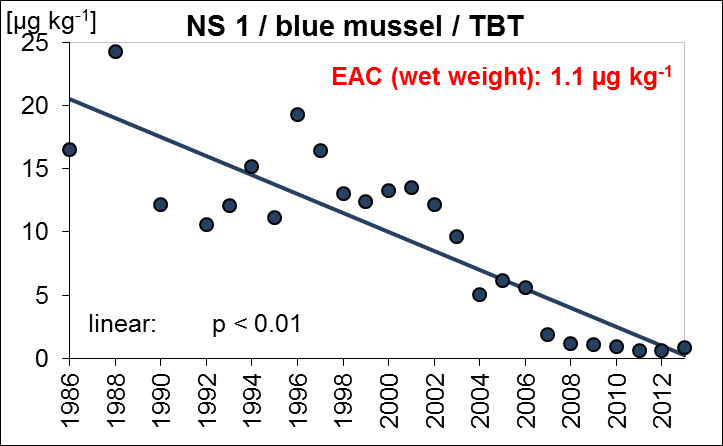

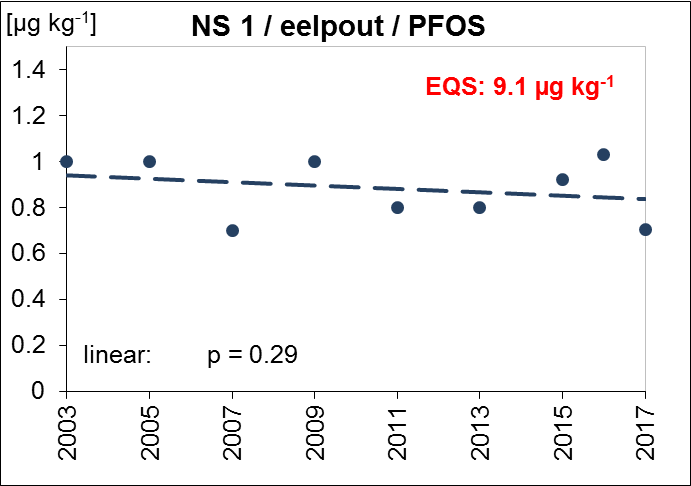

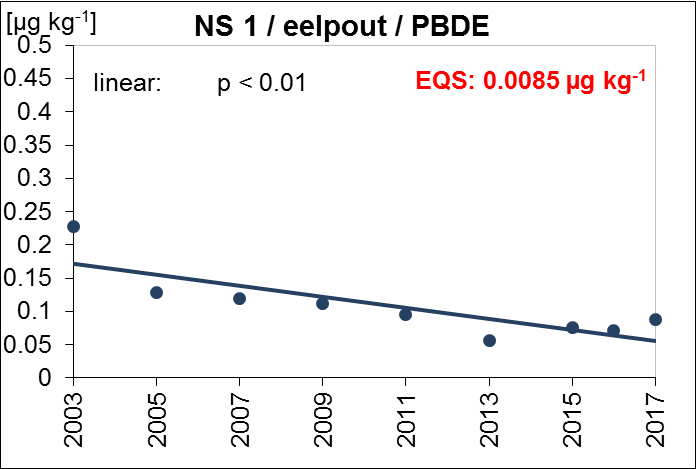

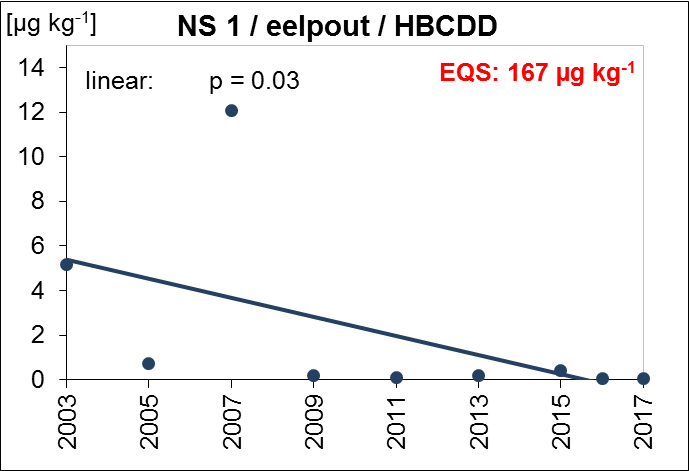

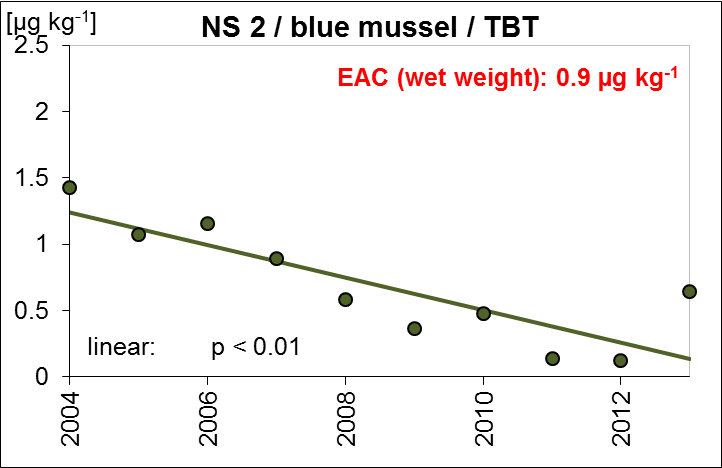

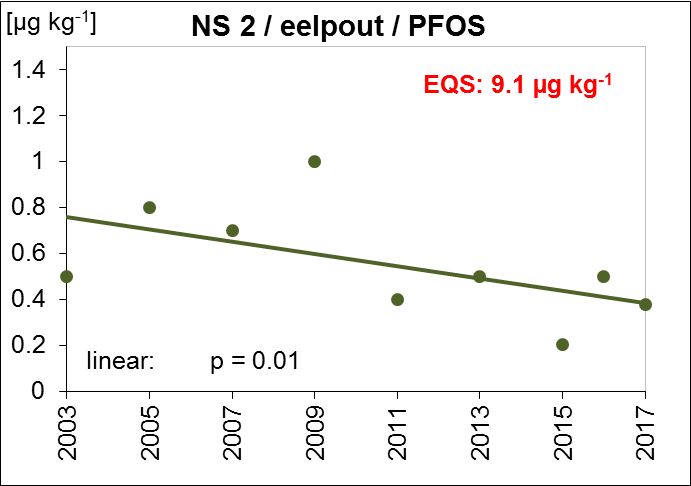

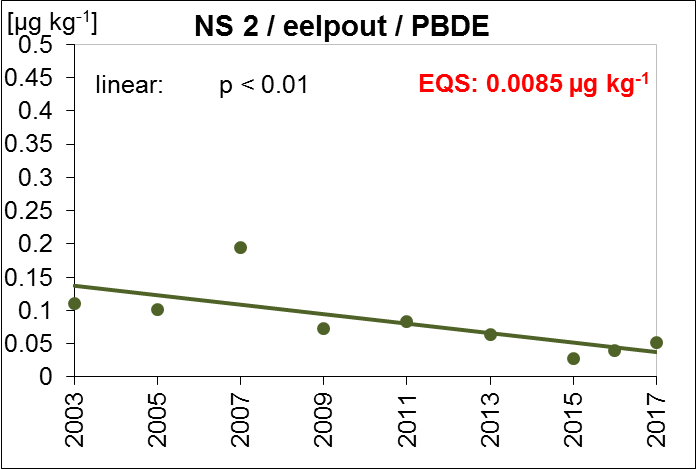

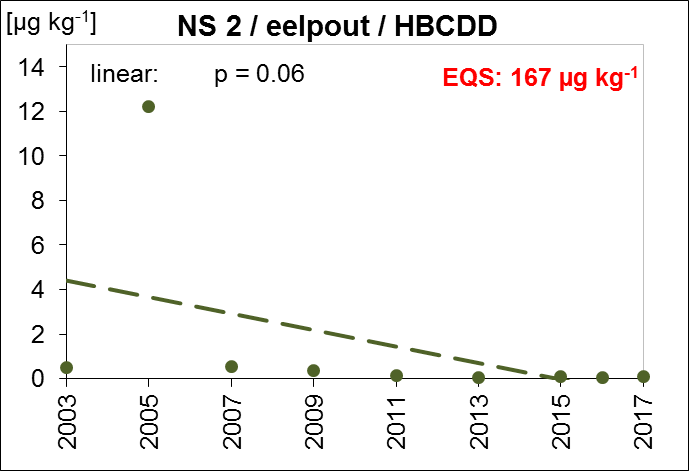


Fig. S4: Temporal trends of TBT in blue mussels (*Mytilus edulis*) and PFOS, PBDE, and HBCDD in eelpout (*Zoarces viviparous,* fillet) from the ESB sampling sites in the North Sea.
Blue: Lower Saxony Wadden Sea, sampling site NS 1; Green: Schleswig-Holstein Wadden Sea, sampling site NS 2. The lines represent the results of the linear regression (solid for significant linear trend, dashed for not significant). EAC: environmental assessment criterion (OSPAR 2004); EQS: environmental quality standard (EC 2013)


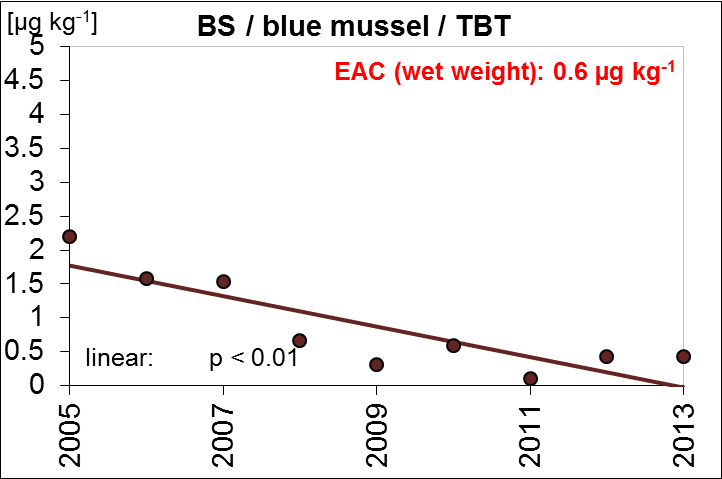

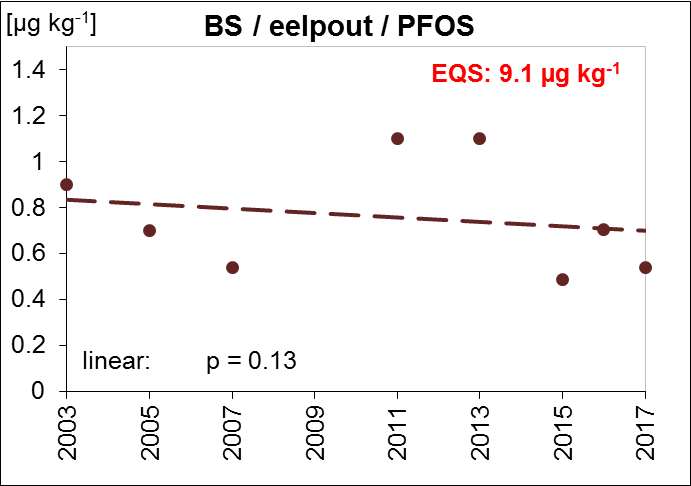

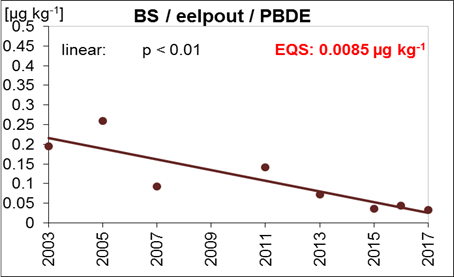

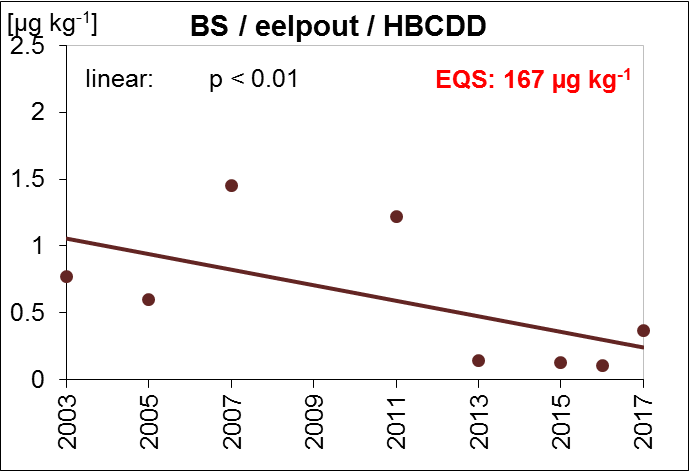


Fig. S5: Temporal trends of TBT in blue mussels (*Mytilus edulis*) and PBDE, HBCDD and PFOS in eelpout (*Zoarces viviparous*, fillet) from the ESB sampling sites in the Baltic Sea / Bodden National Park of Western Pomerania. The lines represent the results of the linear regression (solid for significant linear trend, dashed for not significant).
EAC: environmental assessment criterion (OSPAR 2004); EQS: environmental quality standard (EC 2013)

**References**

BVL (2013) Handbuch Monitoring 2013. Bundesamt für Verbraucherschutz und Lebensmittelsicherheit. Pp 248
[https://www.bvl.bund.de/SharedDocs/Downloads/01_Lebensmittel/01_lm_mon_dokumente/03_Monitoring_Handbuecher/archiv/lm_monitoring_handbuch_2013.html] (accessed 02/2018)

BVL (2017) Handbuch Monitoring 2017. Bundesamt für Verbraucherschutz und Lebensmittelsicherheit. Pp 288
[http://www.bvl.bund.de/SharedDocs/Downloads/01_Lebensmittel/01_lm_mon_dokumente/03_Monitoring_Handbuecher/lm_monitoring_handbuch_2017.pdf?__blob=publicationFile&v=18] (accessed 02/2018)

EC (2006) Commission Regulation (EC) No 1881/2006 of 19 December 2006 setting maximum levels for certain contaminants in foodstuffs. OJ L 364/5. [http://eur-lex.europa.eu/legal-content/EN/TXT/PDF/?uri=CELEX:32006R1881&from=EN] (accessed 02/2018)

EC (2007) Commission Regulation (EC) No 333/2007 of 28 March 2007 laying down the methods of sampling and analysis for the official control of the levels of lead, cadmium, mercury, inorganic tin, 3-MCPD and benzo(a)pyrene in foodstuffs. OJ L88/29. [http://eur-lex.europa.eu/LexUriServ/LexUriServ.do?uri=OJ:L:2007:088:0029:0038:EN:PDF] (accessed 02/2018)

EC (2011) Commission regulation (EU) No 836/2011 of 19 August 2011 amending Regulation (EC) No 333/2007 laying down the methods of sampling and analysis for the official control of the levels of lead, cadmium, mercury, inorganic tin, 3-MCPD and benzo(a)pyrene in foodstuffs. OJ L215/9.[http://eur-lex.europa.eu/legal-content/EN/TXT/PDF/?uri=CELEX:32011R0836&from=DE] (accessed 02/2018)

EC (2013) Directive 2013/39/EU of the European Parliament and of the Council of 12 August 2013 amending Directives 2000/60/EC and 2008/105/EC as regards priority substances in the field of water policy. OJ L226/1. [http://eur-lex.europa.eu/LexUriServ/LexUriServ.do?uri=OJ:L:2013:226:0001:0017:EN:PDF] (accessed 02/2018)

EC (2014) Commission Recommendation (EU) 2014/118 of 3 March 2014 on the monitoring of traces of brominated flame retardants in food. OJ L 65, 5.3.2014, p. 39–40.[http://eur-lex.europa.eu/legal-content/EN/TXT/PDF/?uri=CELEX:32014H0118&from=EN] (accessed 02/2018)

EC (2016) Commission Regulation (EU) 2016/582 of 15 April 2016 amending Regulation (EC) No 333/2007 as regards the analysis of inorganic arsenic, lead and polycyclic aromatic hydrocarbons and certain performance criteria for analysis. OJ L 101/3, 16.4.2016
[http://eur-lex.europa.eu/legal-content/EN/TXT/PDF/?uri=CELEX:32016R0582&from=EN] (accessed 02/2018)

EC (2017) Commission Regulation (EU) 2017/644 of 5 April 2017 laying down methods of sampling and analysis for the control of levels of dioxins, dioxin-like PCBs and non-dioxin-like PCBs in certain foodstuffs and repealing Regulation (EU) No 589/2014. OJ L92/9 [http://eur-lex.europa.eu/legal-content/EN/TXT/PDF/?uri=CELEX:32017R0644&from=EN] (accessed 02/2018)

Horwitz W, Albert R (2006) The Horwitz Ratio (HorRat): A useful index of method performance with respect to precision. J. AOAC Int 89:1095-1109.

Horwitz W, Kamps LR, Boyer KW (1980) Quality assurance in the analysis of foods and trace constituents. J Assoc Off Analy Chem 63: 1344-1354

OSPAR (2004) OSPAR/ICES Workshop on the evaluation and update of background reference concentrations (B/RCs) and ecotoxicological assessment criteria (EACs) and how these assessment tools should be used in assessing contaminants in water, sediment and biota. Report. Le Hague 9-13. February 2004 [https://www.ospar.org/documents?v=6989] (accessed 02/2018)

Swartenbroux F, Albajedo B, Angelidis M, Aulne M, Bartkevics V, Besada V, Bignert A, Bitterhof A, Hallikainen A, Hoogenboom R, Jorhem L, Jud M, Law R, Licht Cederberg D, McGovern E, Miniero R, Schneider R, Velikova V, Verstraete F, Vinas L, Vlad S (2010) Marine Strategy Framework Directive – Task Group 9 Report Contaminants in fish and other seafood. EUR – Scientific and Technical Research series, Luxembourg, JRC/ICES, EUR 24339 EN, Pp 36. [http://publications.jrc.ec.europa.eu/repository/bitstream/JRC58103/tg9%20report%20final_vii.pdf] (accessed 02/2018)

Thompson M (2000) Recent trends in inter-laboratory precision at ppb and sub-ppb concentrations in relation to fitness for purpose criteria in proficiency testing. Analyst 125: 385-386

Webster L, Tronczynski J, Bersuder P, Vorkamp K, Lepom P (2010a) Determination of polybrominated diphenyl ethers (PBDEs) in sediment and biota. ICES Techniques in Marine Environmental Sciences No. 46. 16 pp

Webster L, Bersuder P, Tronczynski J, Vorkamp K, Lepom P (2010b) Determination of Hexabromocyclododecane (HBCD) in sediment and biota. ICES Techniques in Marine Environmental Sciences No. 44. 15 pp
